# Supplementary material for: Second Trimester Abortion: A Dilation and Evacuation Simulation for Gynecologic Surgery and Obstetrics Residents
Source: MedEdPORTAL. 2025 Jan 21;21:11489. doi: 10.15766/mep_2374-8265.11489 (PMC11753717; doi:10.15766/mep_2374-8265.11489)
Supplement: Supplementary file 1 — Materials and Instructions.docxFacilitator Guide.docxLearner Grading Rubric.docxSimulation Debrief.pptxSpeaker Notes for Debrief.docxPre- and Postsimulation Assessment.docxSimulation Video.movFacilitator Sequence of Events.docx [file mep_2374-8265.11489-s001.zip › B. Facilitator Guide.docx]

**Facilitator Guide**

Included is a list of prompts for the facilitator to guide the learners through the following:

- five minute discussion before the simulation regarding preoperative considerations

- step-by-step instructions of the steps of a dilation and evacuation using the uterine and fetal models

- five minute discussion following the simulation about postoperative considerations and complications

**Before the Simulation Discussion**

**Discussion with learners regarding preoperative considerations:*

- How should the patient be positioned for a D&E?
  - Dorsal Lithotomy
- What fetal measurement can help predict necessary cervical dilation?
  - Biparietal Diameter
- What are the benefits to locating the placenta prior to procedure?
  - Surgical planning, diagnosing a previa
- What are the key components of informed consent for a D&E?
  - Description and indications for the procedure
  - Benefits: evacuate products of conception from the uterus
  - Risks: pain, bleeding/hemorrhage, infection, perforation, injury to the cervix, retained placenta, unplanned surgical procedure including laparotomy/hysterectomy
  - Discuss alternative management options (medival vs surgical)
  - If indicated, discuss alternative pregnancy options
- What options are available for anesthesia?
  - Conscious sedation (can be given by a nurse under the direction of a trained family planning provider)
  - Monitored anesthesia care (requires an anesthesia provider)
  - Paracervical block (visualize the cervix and inject 1-2 mL of Lidocaine 1% at the 12, 2, 4, 8, and 10 o’clock positions)
- How is the cervix cleansed prior to the procedure?
  - An antiseptic solution is used to cleanse the cervix in circular motions using a sponge stick or vaginal preparation kit
- What are methods of cervical preparation?
  - Chemical: buccal cytotec 400mcg preop (CAN give with prior uterine scar), consider adding mifepristone 200mg PO after dilator placement prior to D&E if >19 weeks
  - Mechanical: Osmotic dilators include laminaria and dilapan. Laminaria are 2 to 10mm and 60mm in depth. Dilapan are 3-4mm and 55-65mm in depth and are more expensive. Using gel, place mechanical dilators until a snug fit is achieved. Place vaginal gauze if needed to keep dilators in place. Keep track of the number of dilators placed.
- What are optional ancillary clinic tests to offer the patient?
  - Pregnancy Tests: amniocentesis, karyotype, TORCH testing
  - Patient Tests: STI testing
- What are options for antibiotics to use for the procedure?
  - Doxycycline 100mg PO (given 1 hour prior to the procedure and 200mg after the procedure)
  - Alternatives: Azithromycin 500mg IV or PO OR metronidazole PO 500mg BID x 5 days
- What are some speculum options and what are the advantages of different types?
  - A weighted speculum can be helpful for larger fetal parts and forceps angulation
- What are types of large cervical dilators?
  - Pratt Dilators (13 to 59 F)
  - Hegar Dilators (up to 17mm)
- Describe the “no-touch” technique used for cervical dilation?
  - Instruments that enter the uterine cavity should not touch gloved hands or the patient’s skin or vagina
- How much cervical dilation is needed for various gestational ages?
  - The gestational in weeks roughly correlates with the amount of cervical dilation required in millimeters. For example, a fetus at 20 weeks gestation requires at 20mm (or 2 cm) or dilation
- What are some tenaculum options?
  - Ring forceps (less trauma but softer grip)
  - Long Allis
  - Standard tenaculum
- What are types of extraction forceps?
  - Ringed forceps: best for early second trimester
  - Sopher forceps: no pelvic curve
  - Bierer forceps: sharply serrated, pelvic curve, best for late second trimester
- What are the sizes of suction tubing and appropriate aspirating cannula sizes for each?
- Small suction tubing is ⅜ of an inch, compatible with aspiration cannulas < 16mm
- Large suction tubing is ½ of an inch, compatible with aspiration cannulas >/= 16mm
- What are options for uterotonics?
- Methylergonovine (Methergine) is first line uterotonic for an earlier gestational age
- Misoprostol (Cytotec) is another option but has a slower onset of action
- Consideration of Pitocin is appropriate if >20 weeks and after extraction of the fetus
- Carbaprost (Hemabate) can be considered but it is not a first line agent

**Simulation Step by step instructions: Use of the model**

*Procedural Steps*

1. The learner should begin by performing the initial exam.
   1. Assess position of uterus
   2. Verbalize to remove dilators if necessary
   3. Assess cervical dilation
2. The learner should verbalize placement of the speculum and cleansing cervix with an antiseptic solution.
3. Next the learner should place the instrument for the tenaculum on the anterior lip of the cervix (the anterior portion of the rubber of the water container lip).
4. Ask the learner to verbalize dilation of the cervix.
   1. Emphasize “no touch” technique (refrain from touching tips of dilators, only hold dilators in the middle to mimic procedures performed outside of the sterile operating room).
   2. Ask the learner to explain calculation for size dilator corresponding to desired cervical dilation.
5. Next, the forceps should be used to rupture the membranes.
   1. Discuss with the learner method of either rupturing membrane regardless of fetal position or flipping the fetus to breech prior to rupture to allow for extraction of calvarium last depending on surgeon preference.

| 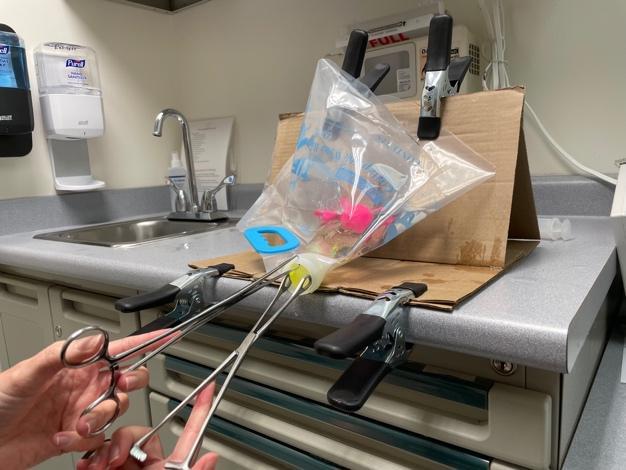 | 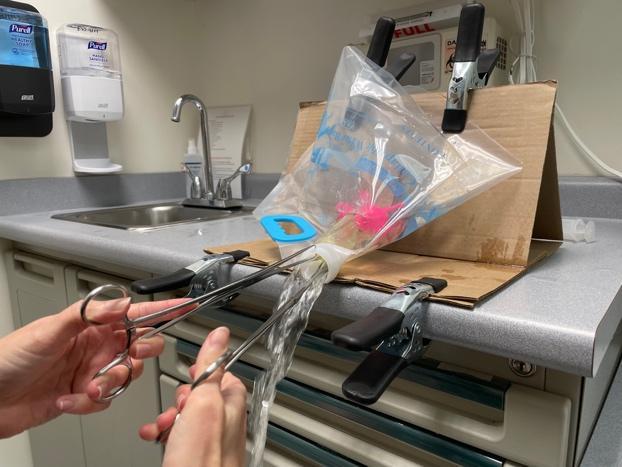 |
| --- | --- |

- 1. Discuss options to rupture membranes with either 16F cannula/suction (if using) or manually with forceps.

1. Next, extract the fetal parts with the forceps.
   1. Grasp the fetal part, withdraw forceps, and rotate to remove while maintaining traction.
   2. Keep track of fetal parts: 2 lower extremities, 2 upper extremities, rib cage/spine, calvarium.

| 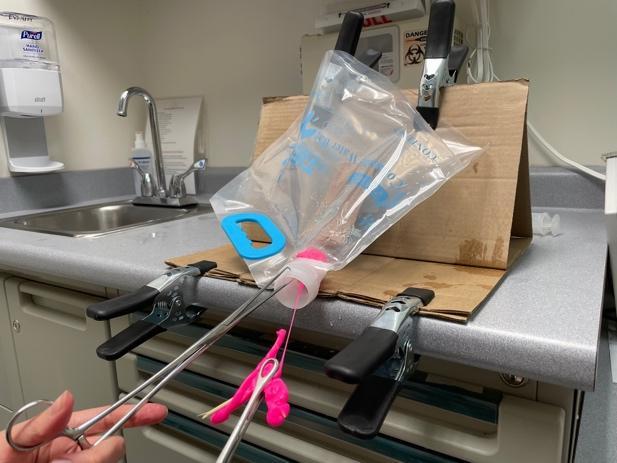 | 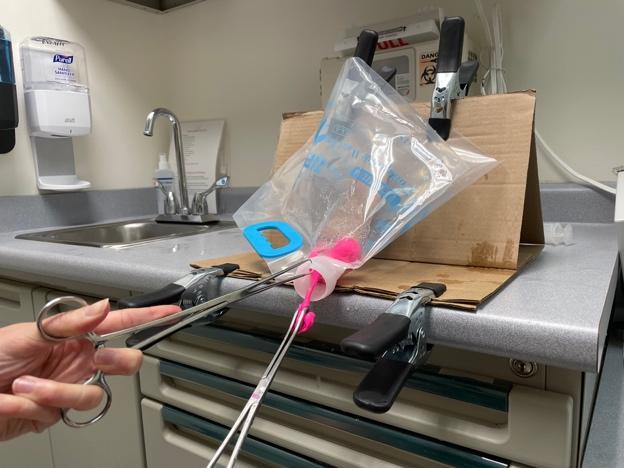 |
| --- | --- |

- 1. Compress the calvarium prior to removal.


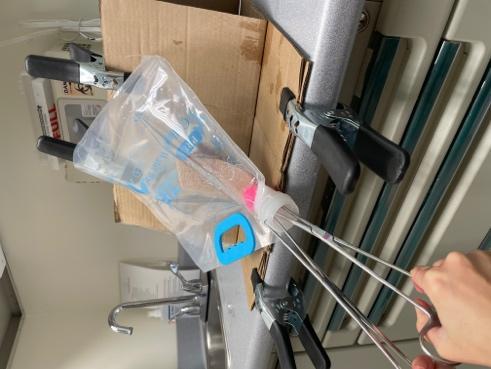


1. Next, use the forceps to remove the placenta.
   1. If difficult, explain the Hanson maneuver of using the nondominant hand on the fundus to guide the curette. (Reference: Paul M, Lichtenberg ES, Borgatta L, Grimes DA, Stubblefield PG, Creinin MD, editors. Management of unintended and abnormal pregnancy: comprehensive abortion care. Oxford: Blackwell Publishing Ltd.; 2009:252–63).
   2.
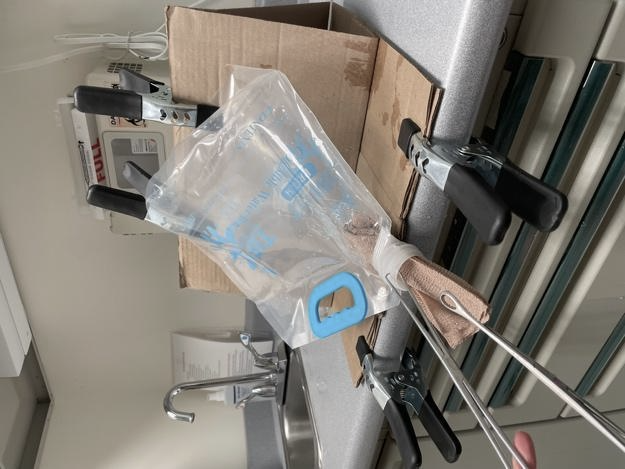

2. Perform a “check” curettage.
   1. Insert (or verbalize using) blunt edge curette to ensure complete evacuation and check for perforation.
3. Perform a suction curettage (*optional*)
   1. Insert (or verbalize using) a 12mm cannula with small suction tubing.
4. Lastly, examine the tissue.
   1. Confirm the presence of chorionic villi or total fetal parts depending on gestational age.
5. For advanced learners, place a sheet over the model or use an opaque water container in order to emphasize performing this procedure based on tactile feel.

**Facilitator’s Guide: After the Simulation Discussion**

**Discussion with learners on post operative considerations:*

- When is it clinically indicated to give Rhogam? What dose? (using Society of Family Planning guidelines. (Horvath S, Goyal V, Traxler S, Prager S. Society of Family Planning committee consensus on Rh testing in early pregnancy. Contraception. 2022 Oct;114:1-5. doi: 10.1016/j.contraception.2022.07.002. Epub 2022 Jul 21. PMID: 35872236.)
  - No administration required < 12 weeks for SAB or IAB
  - Administer 50mcg if <12 weeks for sharp curette, ectopic pregnancy, or other invasive procedures
  - Administer 100mcg between 13 to 18 weeks
  - Administer 300mcg if >18 weeks
- How should patients be counseled about contraception after the procedure? What options are available?
  - Patients should be informed that ovulation can occur as early as 21 days after the procedure (with some reports as early as 10 days)
  - All contraception options are available for same day initiation except for cervical caps/diaphragms which require the complete involution of the uterus
- How should patients be counseled about lactation? What are management options?
  - Patients should be informed that lactation can occur after second trimester procedures and can be managed conservatively (NSAIDs, ice packs, and avoiding breast stimulation)

**Discussion with learners on post operative complications:*

- How should retained tissue be managed?
  - For a small amount, administering prostaglandins is appropriate
  - For a large amount of tissue, repeating aspiration is necessary, which would require redosing the antibiotics
- How should a post abortion infection be managed?
  - Post abortion infections should be treated with antibiotics and evacuating the uterus, but avoiding sharp curettage
  - First line antibiotics are Ampicillin AND Gentamicin AND Clindamycin. Alternatively, patients can receive Ceftriaxone AND Doxycycline AND Flagyl
- How should hematometra be managed?
  - For retained blood in the uterine cavity, repeat suction dilation and curettage. Antibiotics should be re-dosed if sharp curettage is used
- How should a post-abortion hemorrhage be treated?
  - Treat the underlying cause:
    - Atony: Bimanual massage, uterotonics, intrauterine balloon, uterine artery embolization, hysterectomy
    - Laceration: Identification and repair
    - Retained Tissue: Adequate evacuation of uterus; curettage
    - Coagulopathies: Laboratory evaluation
- How can a cervical laceration be prevented?
  - Prevent a cervical laceration with adequate cervical dilation and preparation.
  - For non-emergent cases without adequate cervical preparation or dilation, consider further preparation and delaying the procedure
- What are tools to help identify a uterine perforation?
  - Vaginal or rectal examination for excessive bleeding or defects
  - Ultrasound
  - Hysteroscopy, Cystoscopy, or Laparoscopy
