# Supplementary material for: Second Trimester Abortion: A Dilation and Evacuation Simulation for Gynecologic Surgery and Obstetrics Residents
Source: MedEdPORTAL. 2025 Jan 21;21:11489. doi: 10.15766/mep_2374-8265.11489 (PMC11753717; doi:10.15766/mep_2374-8265.11489)
Supplement: Supplementary file 1 — Materials and Instructions.docxFacilitator Guide.docxLearner Grading Rubric.docxSimulation Debrief.pptxSpeaker Notes for Debrief.docxPre- and Postsimulation Assessment.docxSimulation Video.movFacilitator Sequence of Events.docx [file mep_2374-8265.11489-s001.zip › C. Learner Grading Rubric.docx]

**Learner Grading Rubric**

Designed for use by the facilitator to assess learners on their ability to perform the steps of a dilation and evacuation

| **During the simulation, does the learner:** | **Yes** | **Yes**  *(with prompting)* | **No** |
| --- | --- | --- | --- |
| Describe how to position the patient for this procedure |  |  |  |
| Verbalize how to cleanse the cervix prior to the procedure |  |  |  |
| Describe how to perform a paracervical block |  |  |  |
| Correctly verbalize how to dilate a cervix using the “no-touch” technique |  |  |  |
| State how much cervical dilation is needed based on the fetus’s measured gestational age |  |  |  |
| Rupture amniotic fluid |  |  |  |
| Disarticulate fetal limbs to extract fetus using a gentle, rotational maneuver |  |  |  |
| Compress fetal calvarium prior to removal |  |  |  |
| Count all extremities, thorax/spine, and calvarium to ensure all fetal parts were extracted |  |  |  |
| Remove placenta |  |  |  |
| Inspect tenaculum site |  |  |  |
