# Supplementary material for: Second Trimester Abortion: A Dilation and Evacuation Simulation for Gynecologic Surgery and Obstetrics Residents
Source: MedEdPORTAL. 2025 Jan 21;21:11489. doi: 10.15766/mep_2374-8265.11489 (PMC11753717; doi:10.15766/mep_2374-8265.11489)
Supplement: Supplementary file 1 — Materials and Instructions.docxFacilitator Guide.docxLearner Grading Rubric.docxSimulation Debrief.pptxSpeaker Notes for Debrief.docxPre- and Postsimulation Assessment.docxSimulation Video.movFacilitator Sequence of Events.docx [file mep_2374-8265.11489-s001.zip › A. Materials and Instructions.docx]

**Materials List and instructions for creation of uterine and fetal models**

intended for facilitator, 8-10 minutes set up time for both models

**Materials list** (items listed are intended for 1 iteration)

- 1 Cardboard box (broken down, use small [[4x4x4](https://www.theboxery.com/Product.asp?d=103545&Product=CXMMAI11&Name=Corrugated+Mailers+4x4x4) inch or [8x8x8](https://www.theboxery.com/Product.asp?d=1055&Product=MD888&Name=Multi-Depth+Boxes+8x8x8) inch] or medium size [9x9x9 inch])
- 4 Spring clamps (size used in this model = 4-6” with 2.5” jaw opening)
- 1 pair of Scissors
- 1 roll of Extra-large double sided heavy duty mounting tape (width used in this model = 1.18”)
- 1 roll of Coban Self-Adhesive Wrap (3” x 5 yard roll)
- 1 collapsible water container bag (5L bag and 1L bag used in this model, different spout sizes can mimic various cervical dilations)
- 1 Water balloon (inflation capacity 5 inches; will need multiple depending on number of iterations) single-use
- 1 Tin of Thinking putty (4 inch tin; will need multiple depending on number of iterations)
- 2-3 cm Styrofoam ball, (larger styrofoam balls as needed to simulate varying gestational ages) - single-use
- 2-4 Vaginal swabs (will need multiple depending on number of iterations)
- 30-40 mL Tap water
- 1 Tumi syringe (to instill tap water into balloons)
- 1 pair of Forceps (options include ring forceps, Sopher forceps, and Bierer forceps)
- 1 Under buttock drape or other container to collect fluid
- 1 single tooth Tenaculum

*Optional materials to perform procedure under suction

- 16F suction cannula
- Suction machine
- Nitrile Gloves (single-use)

Total Cost: $73.35

For a video instruction to create the fetus and uterine model, please reference Appendix G. The intent is for learners and facilitators to review the video prior to performing the steps of the D&E. Learners and facilitators can choose to view this video prior to creation of the stimulation if they need further assistance after reviewing the below instructions.

**Step by step instructions: Creation of fetus**

1. Acquire one tin of thinking putty, one 2-3 cm Styrofoam ball, and two vaginal swabs
2. Break the vaginal swab into five 4-5 cm pieces.
3. Mold a quarter sized amount of thinking putty around one vaginal swab piece. This will serve as fetal limbs, spine, and thorax. Repeat this process for all vaginal swabs.


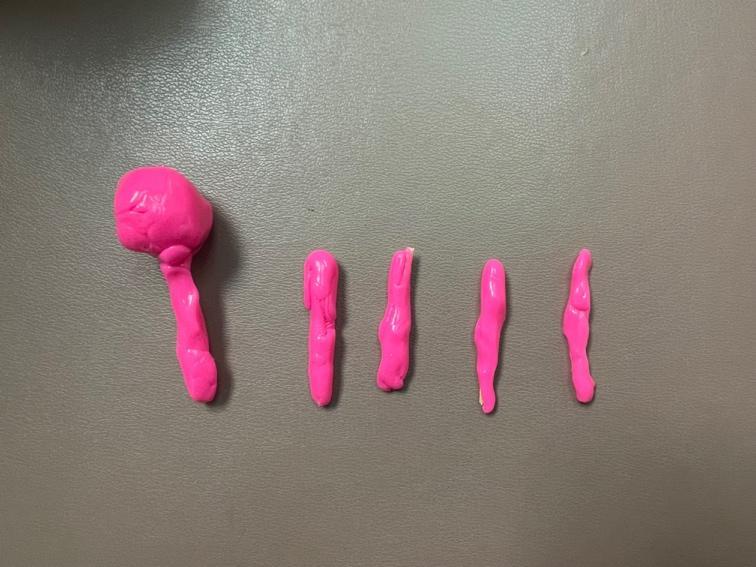


1. Pierce the Styrofoam ball with one vaginal swab to serve as the spine/thorax and calvarium.
2. Mold the remaining vaginal swabs to this central vaginal swab to simulate the fetal arms and legs.
3. Based on year level objectives, you may use longer/shorter vaginal swabs and more/less putty in order to simulate varying gestational ages. Additionally, you may use varying foam sphere sizes to simulate larger/smaller calvariums. For example: regarding length of the fetus, approximately 17 cm corresponds to 15 weeks, 20 cm to 17 weeks, 24 cm to 19 weeks, and 27 cm to 21 weeks. [8].

**Step by step instructions: Creation of uterine model**

1. Assemble materials from the list above.
2. Take the collapsible water container and cut a seam at the top (the furthest from the opening). This will allow for filling/re-filling for each iteration. You may alter the collapsible water container you wish to use based on how much cervical dilation you wish to have. Larger dilations can be helpful to use for new learners who are learning how to perform a D&E. Smaller dilation can simulate more challenging cases.


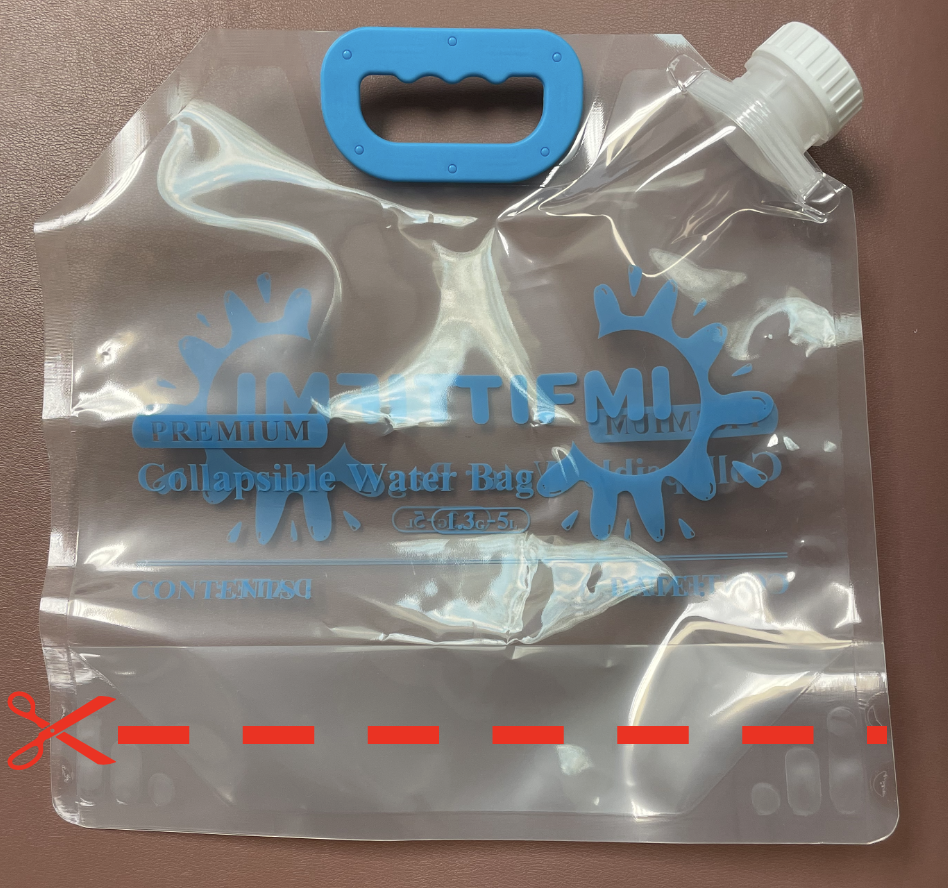


1. Using the Tumi syringe, fill a water balloon with approximately 40-55 cc of water.
   1. Place the water balloon through the water bottle and “wedge” it into the opening of the water container.
2. Cut off a 1 meter segment of coban wrap. Fold the wrap in halves until you have approximately an 8cm segment to mimic the placenta. Use mounting tape to attach it to somewhere within the water container (uterine cavity).

| 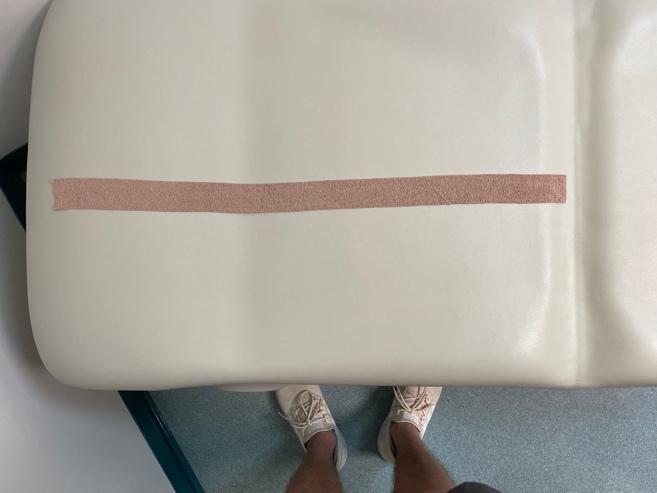 | 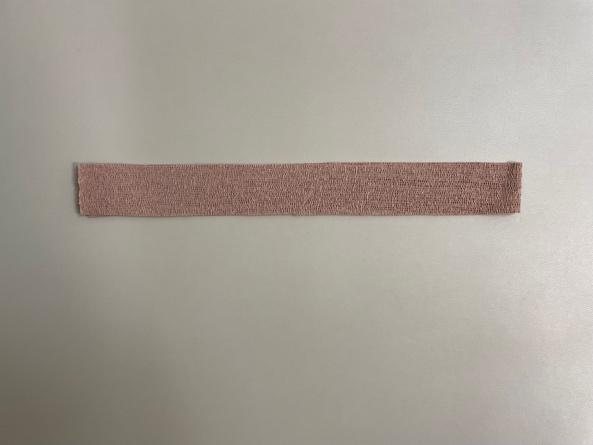 | 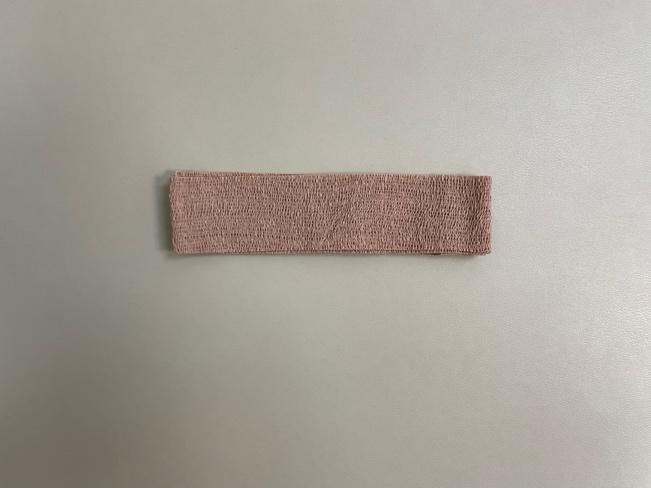 | 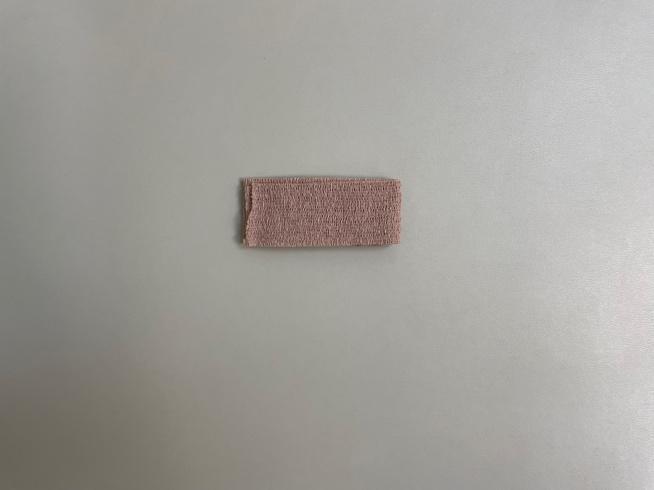 |
| --- | --- | --- | --- |
|  |  |  |  |

1. Insert the simulated fetus into the uterine model.


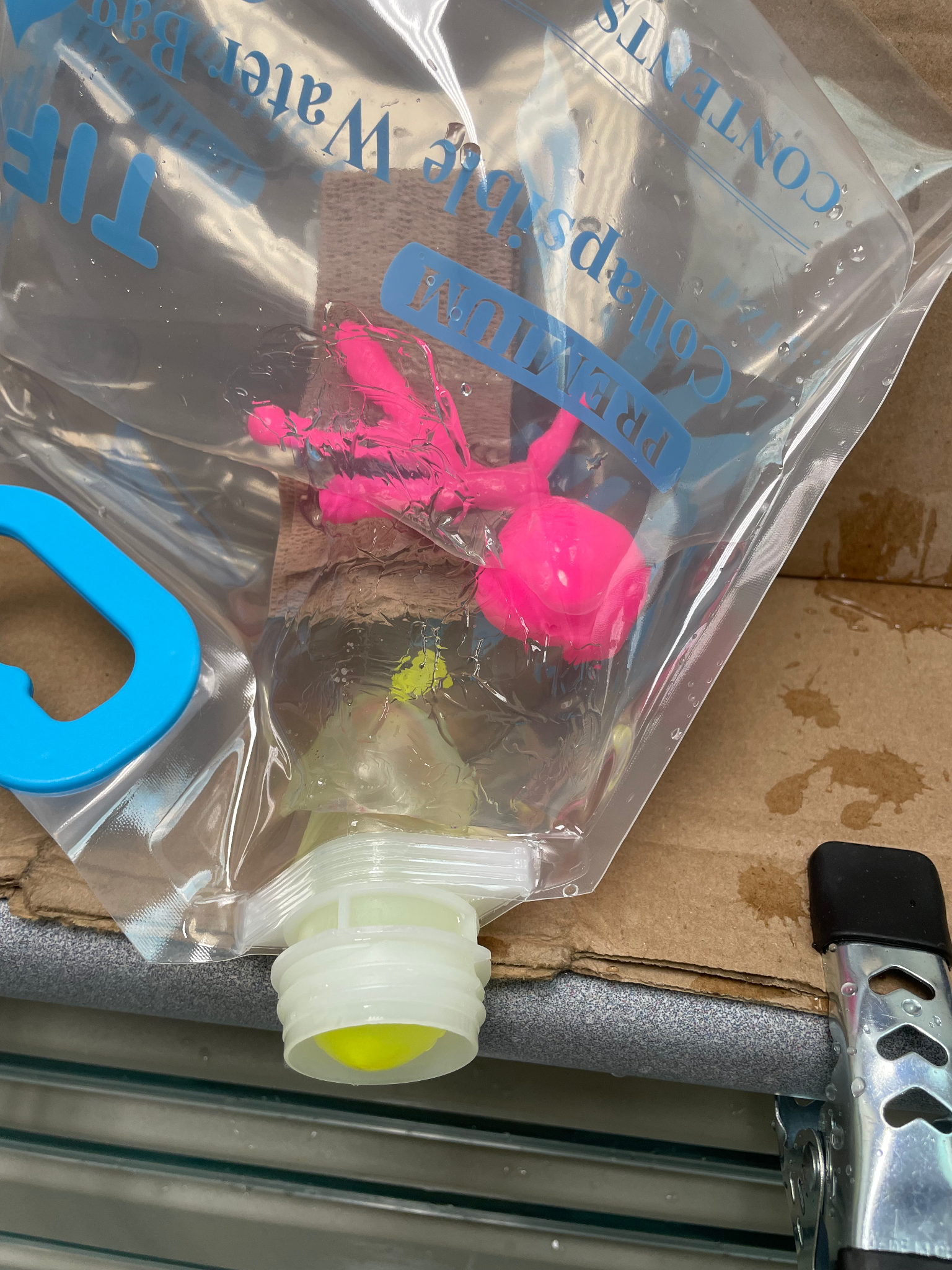


1. Now that the uterine structure has been assembled, mount it to the cardboard and desired training table.
   1. Completely breakdown the cardboard box.


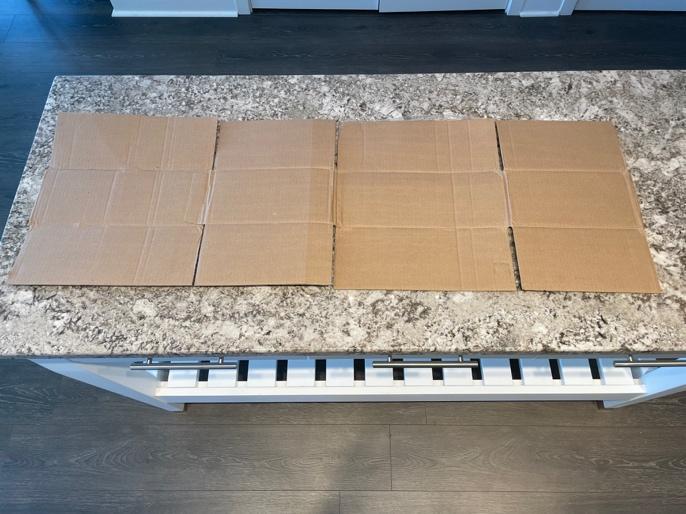


- 1. Fold the cardboard box in half along its natural bend point.


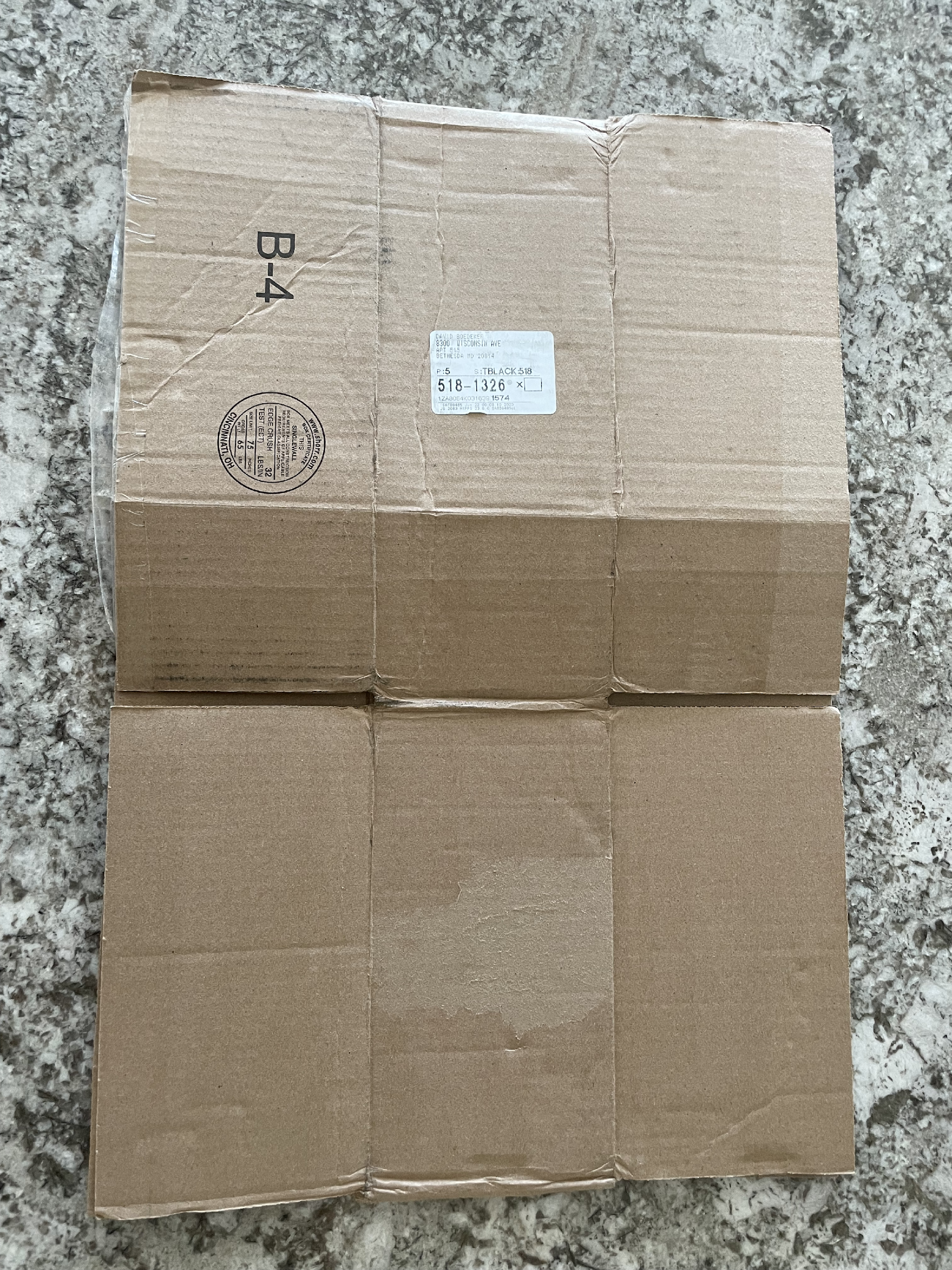


- 1. Note the labeling in the photo below to illustrate the remaining steps.


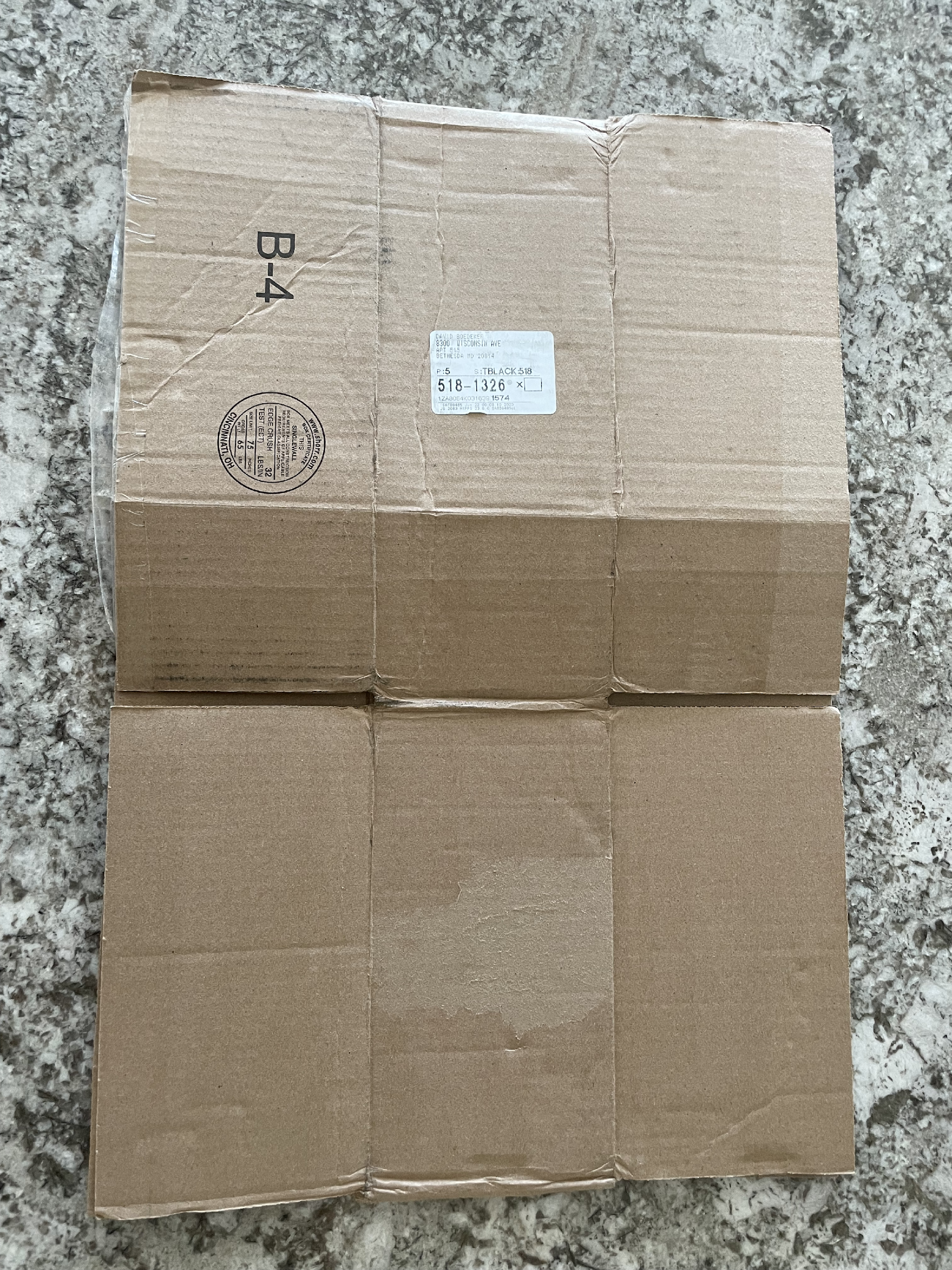


A

B



- 1. Create a crease in side B by folding the side several inches from the box’s natural crease point.

| 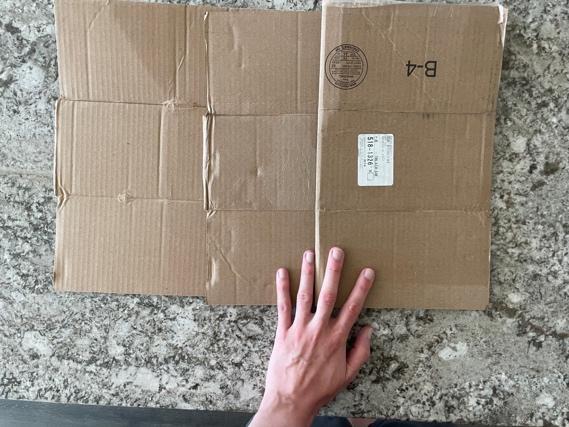 | 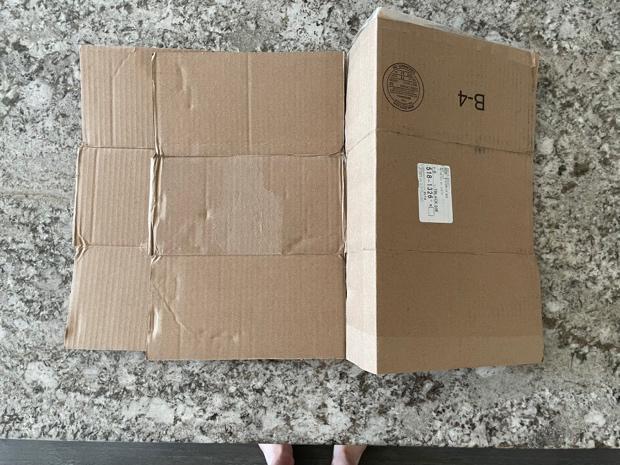 |
| --- | --- |

- 1. By creating this new crease, note the addition of side C in the figure below.

| 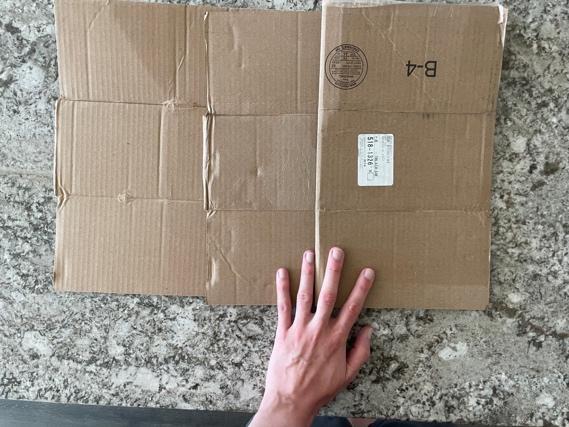  A  B | 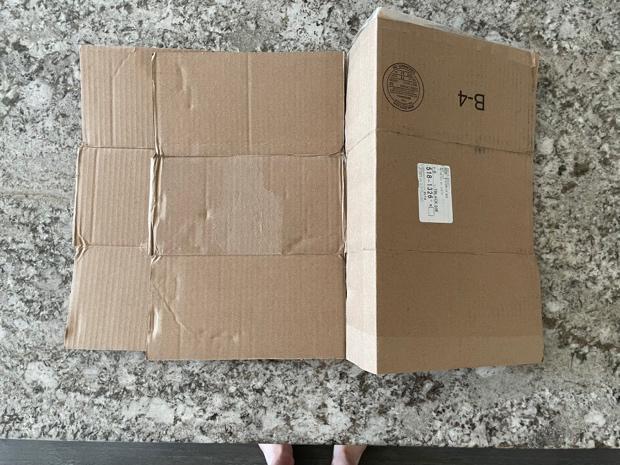  A  C  B |
| --- | --- |

- 1. Rotate side C so it lies on top of side A. Align the free edges of the box together. Side B and side C will create a triangular stand, which will stand perpendicular to side A.


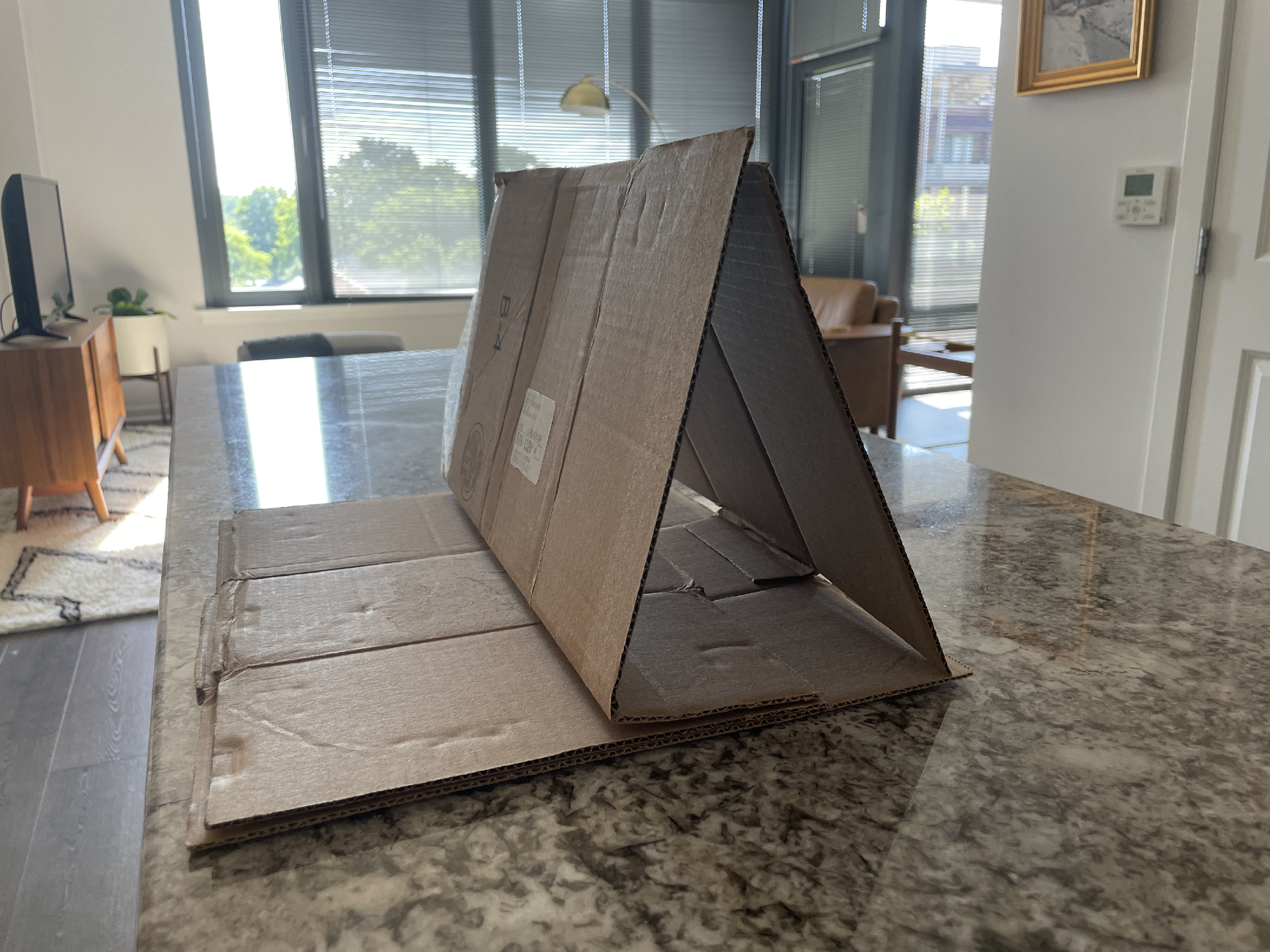


- 1. Now secure the cardboard box to your work surface. Two of the clamps should secure side A (the base) to the table.
  2. The other two clamps should secure/suspend the water bottle to the superior aspect of side B.

| 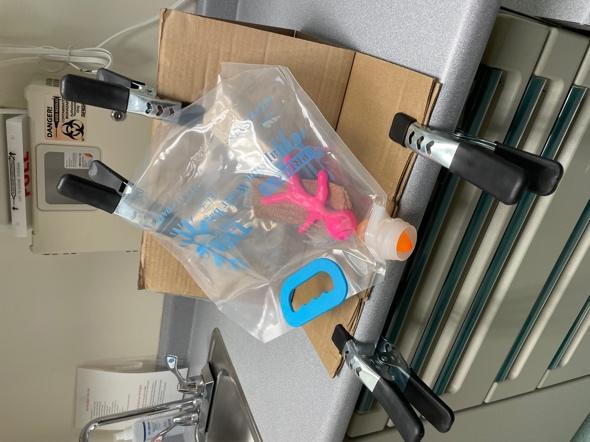 | 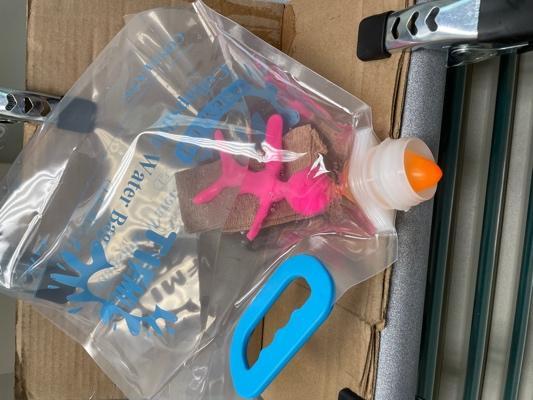 |
| --- | --- |

1. Attach the under buttocks drape to the model using the clamps. Alternatively, place another collection method underneath the mouth of the bottle.
2. Slowly fill the water bottle with water to serve as the amniotic fluid.
