# Supplementary material for: Second Trimester Abortion: A Dilation and Evacuation Simulation for Gynecologic Surgery and Obstetrics Residents
Source: MedEdPORTAL. 2025 Jan 21;21:11489. doi: 10.15766/mep_2374-8265.11489 (PMC11753717; doi:10.15766/mep_2374-8265.11489)
Supplement: Supplementary file 1 — Materials and Instructions.docxFacilitator Guide.docxLearner Grading Rubric.docxSimulation Debrief.pptxSpeaker Notes for Debrief.docxPre- and Postsimulation Assessment.docxSimulation Video.movFacilitator Sequence of Events.docx [file mep_2374-8265.11489-s001.zip › D. Simulation Debrief.pptx]

## Slide 1
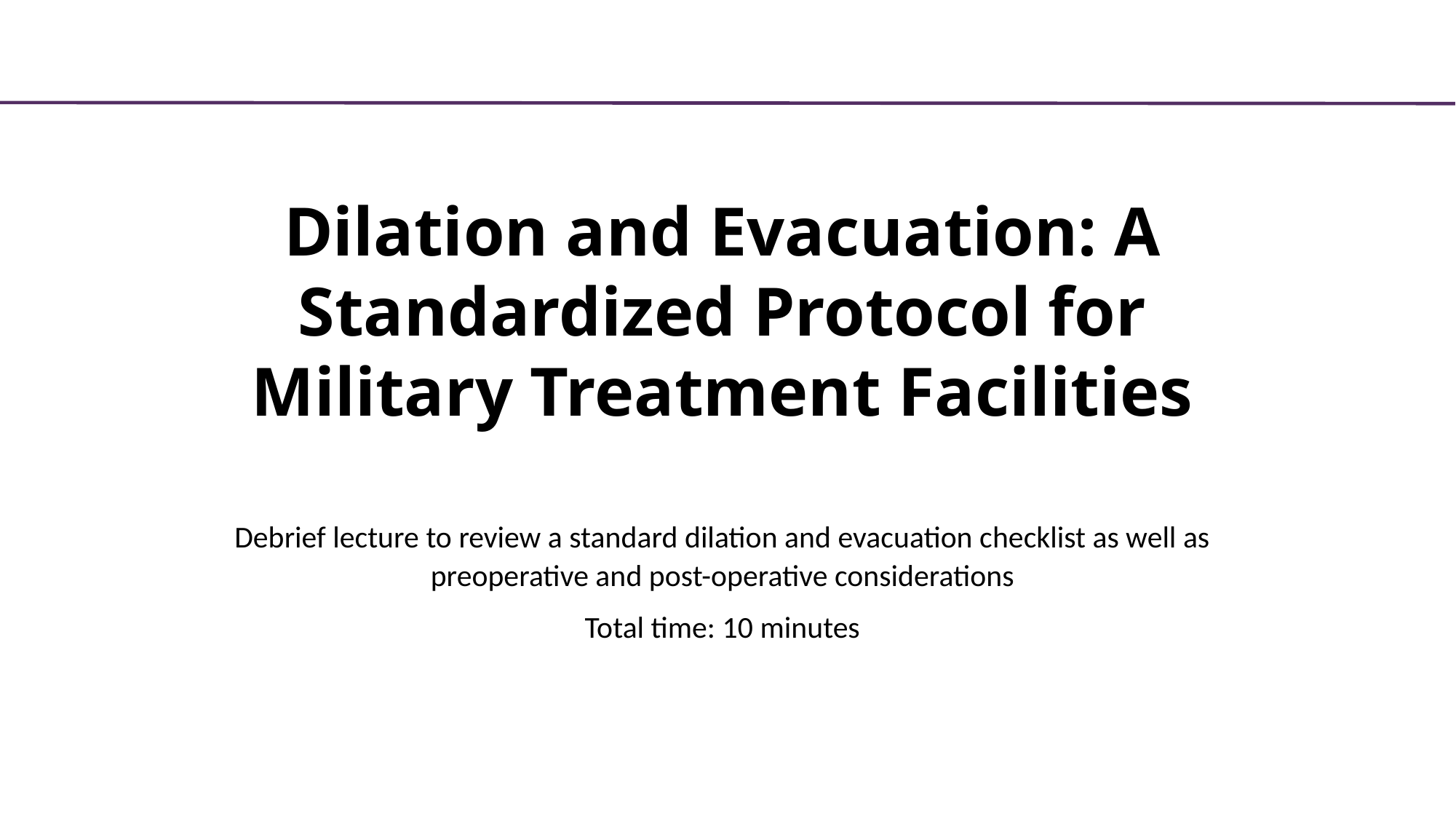

Dilation and Evacuation: A Standardized Protocol for Military Treatment Facilities
Debrief lecture to review a standard dilation and evacuation checklist as well as preoperative and post-operative considerations
Total time: 10 minutes

## Slide 2
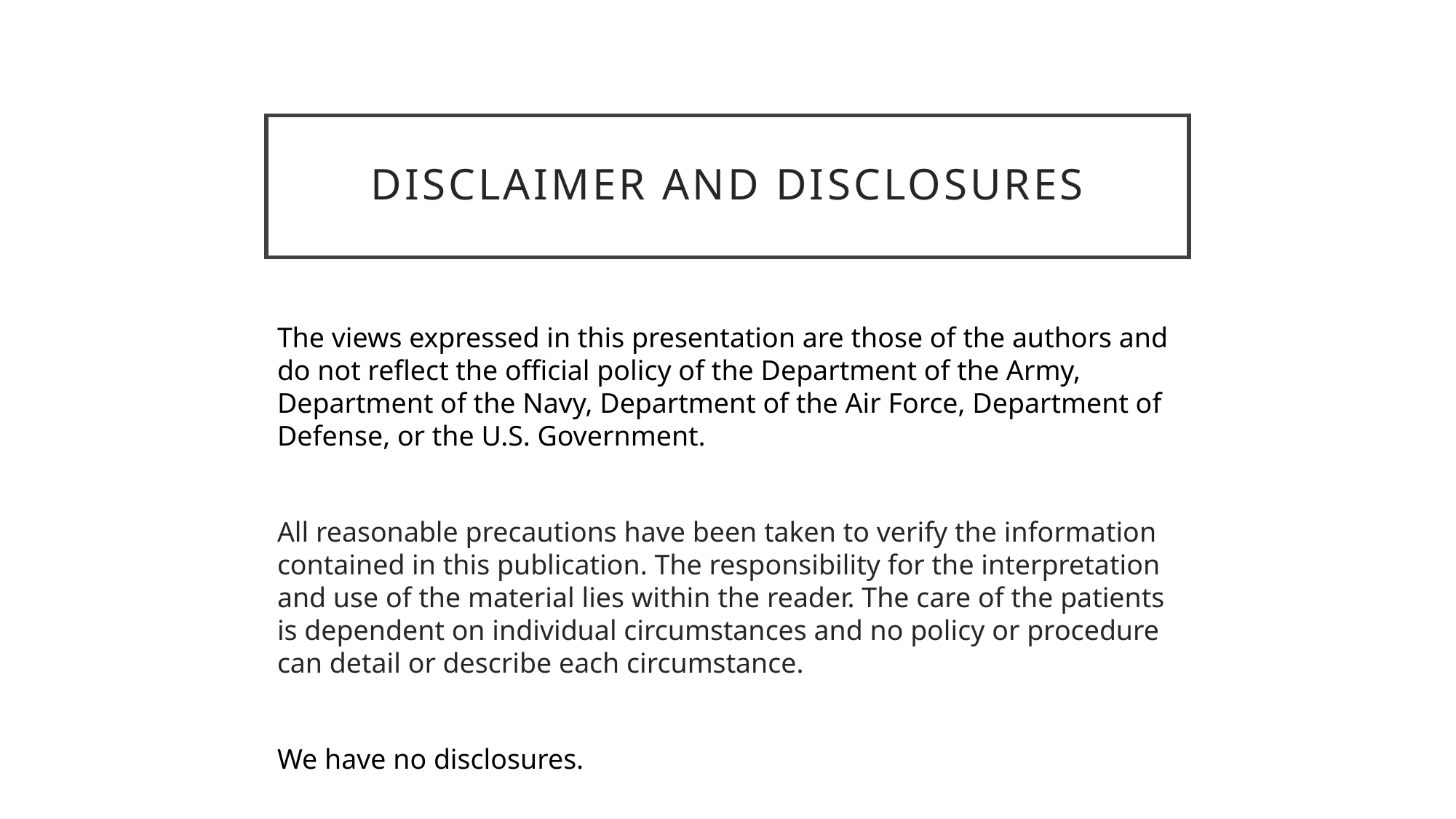

# Disclaimer and Disclosures
The views expressed in this presentation are those of the authors and do not reflect the official policy of the Department of the Army, Department of the Navy, Department of the Air Force, Department of Defense, or the U.S. Government.
All reasonable precautions have been taken to verify the information contained in this publication. The responsibility for the interpretation and use of the material lies within the reader. The care of the patients is dependent on individual circumstances and no policy or procedure can detail or describe each circumstance.
We have no disclosures.

## Slide 3
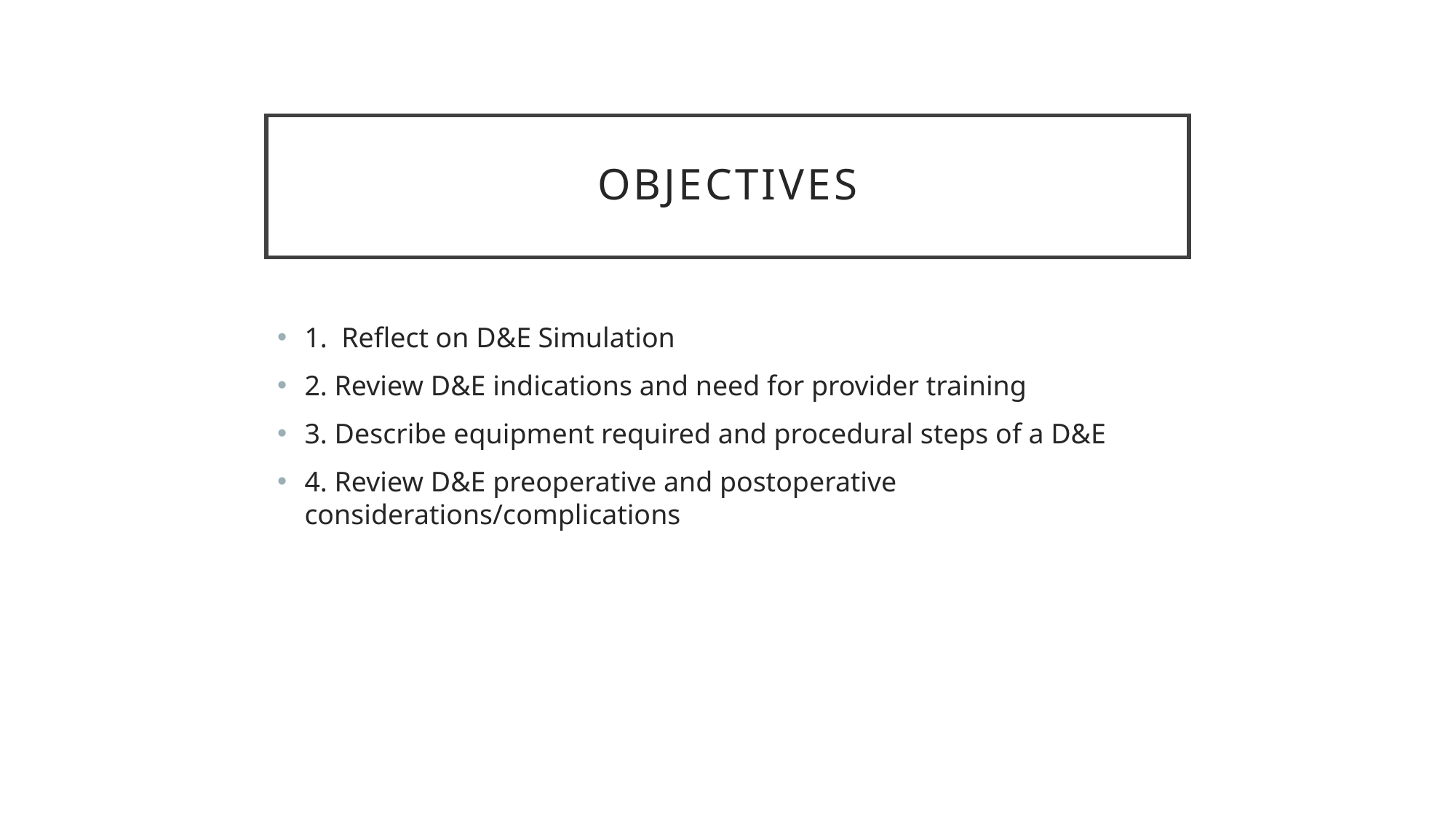

# OBJECTIVES
1. Reflect on D&E Simulation
2. Review D&E indications and need for provider training
3. Describe equipment required and procedural steps of a D&E
4. Review D&E preoperative and postoperative considerations/complications

## Slide 4
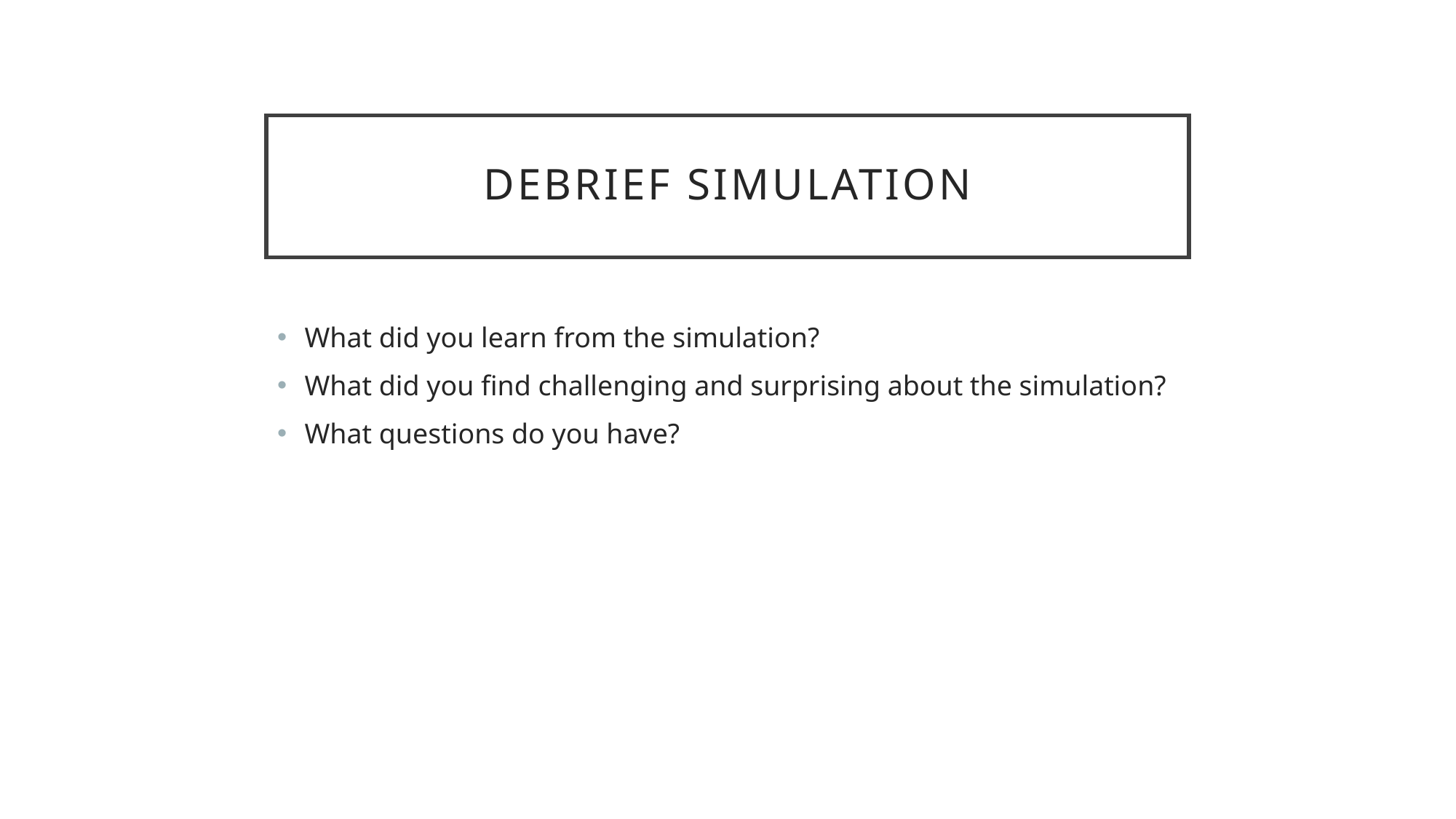

# Debrief simulation
What did you learn from the simulation?
What did you find challenging and surprising about the simulation?
What questions do you have?

## Slide 5
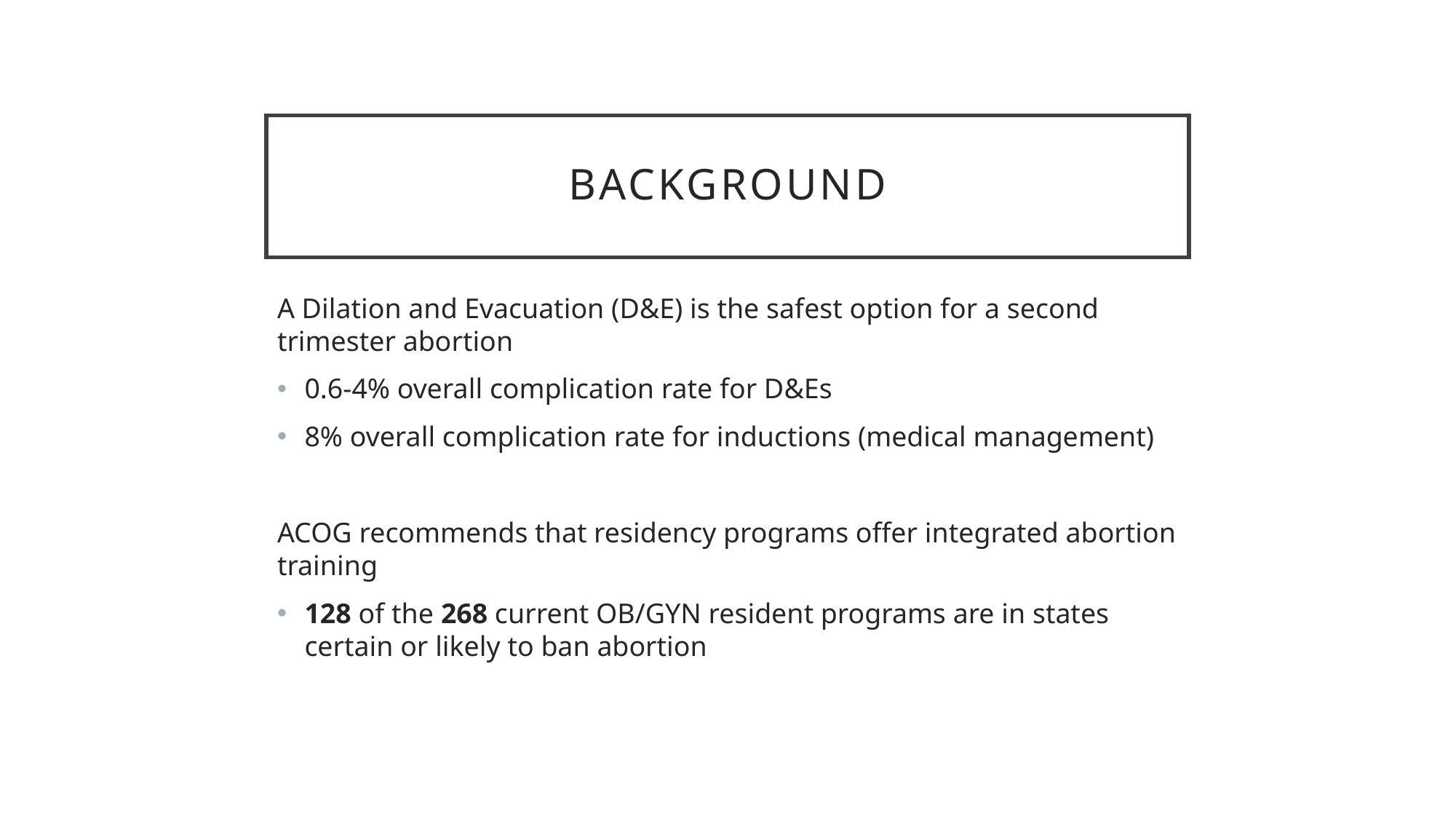

# Background
A Dilation and Evacuation (D&E) is the safest option for a second trimester abortion
0.6-4% overall complication rate for D&Es
8% overall complication rate for inductions (medical management)
ACOG recommends that residency programs offer integrated abortion training
128 of the 268 current OB/GYN resident programs are in states certain or likely to ban abortion

## Slide 6
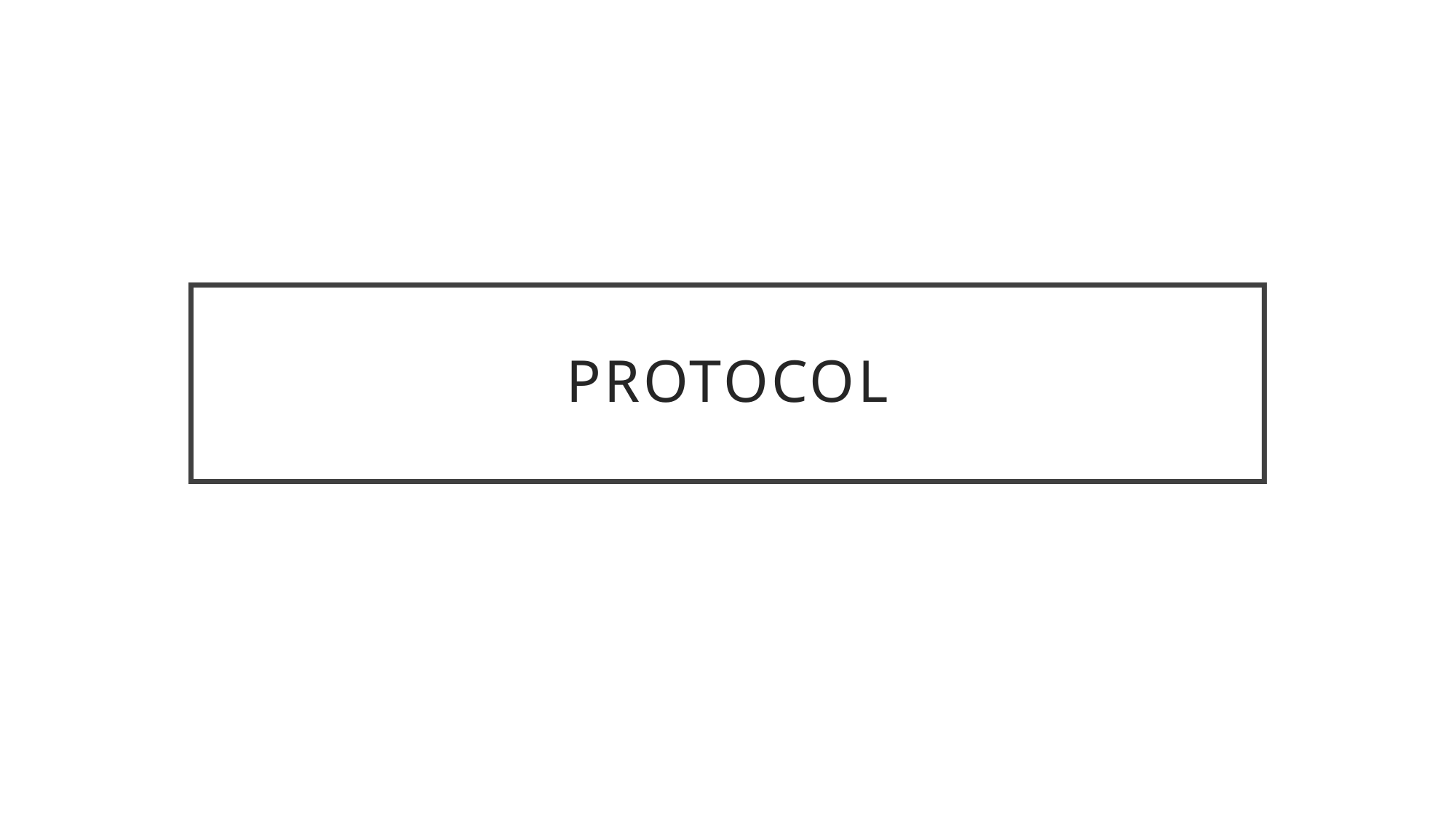

# Protocol

## Slide 7
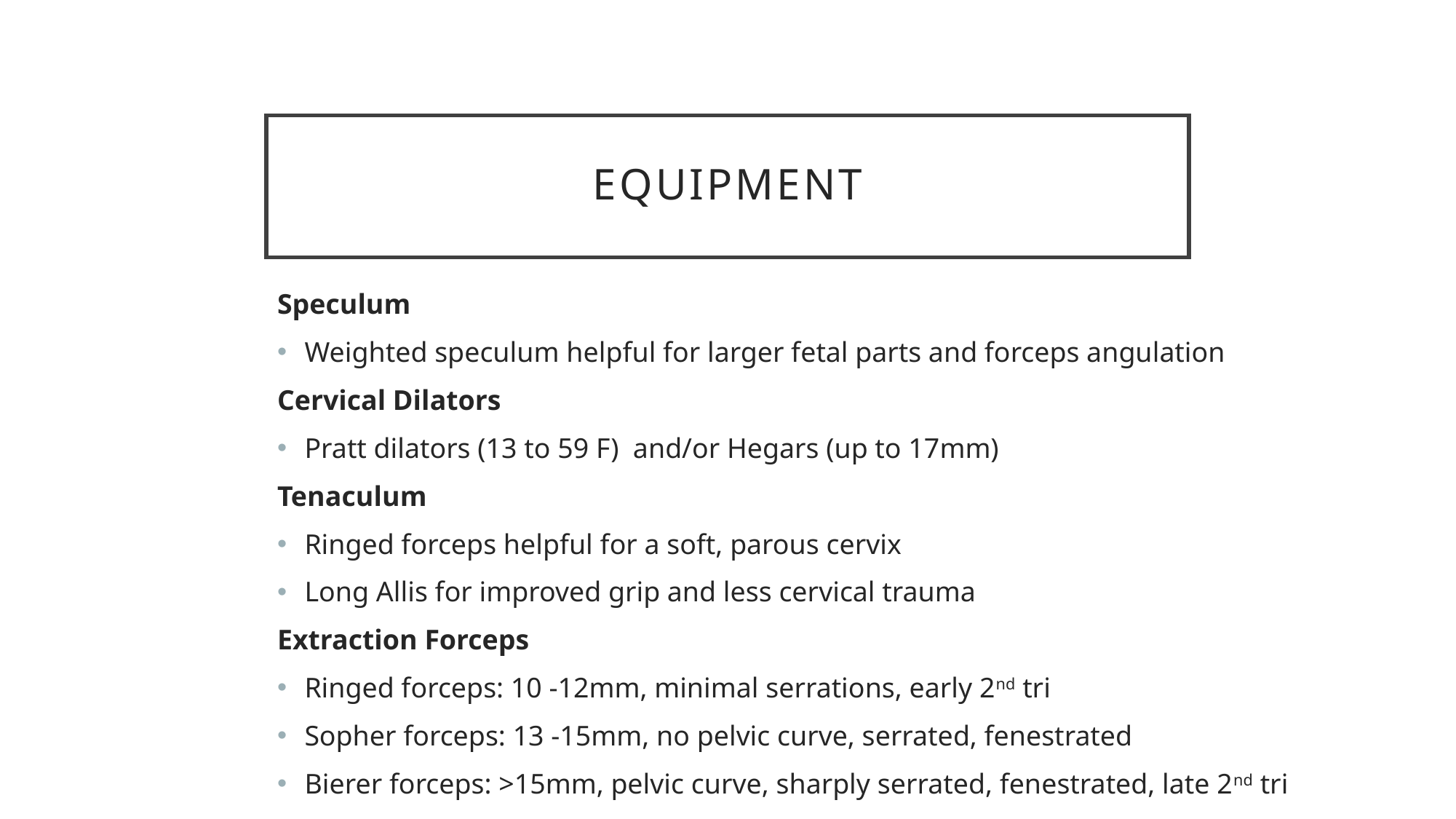

# Equipment
Speculum
Weighted speculum helpful for larger fetal parts and forceps angulation
Cervical Dilators
Pratt dilators (13 to 59 F) and/or Hegars (up to 17mm)
Tenaculum
Ringed forceps helpful for a soft, parous cervix
Long Allis for improved grip and less cervical trauma
Extraction Forceps
Ringed forceps: 10 -12mm, minimal serrations, early 2nd tri
Sopher forceps: 13 -15mm, no pelvic curve, serrated, fenestrated
Bierer forceps: >15mm, pelvic curve, sharply serrated, fenestrated, late 2nd tri

## Slide 8
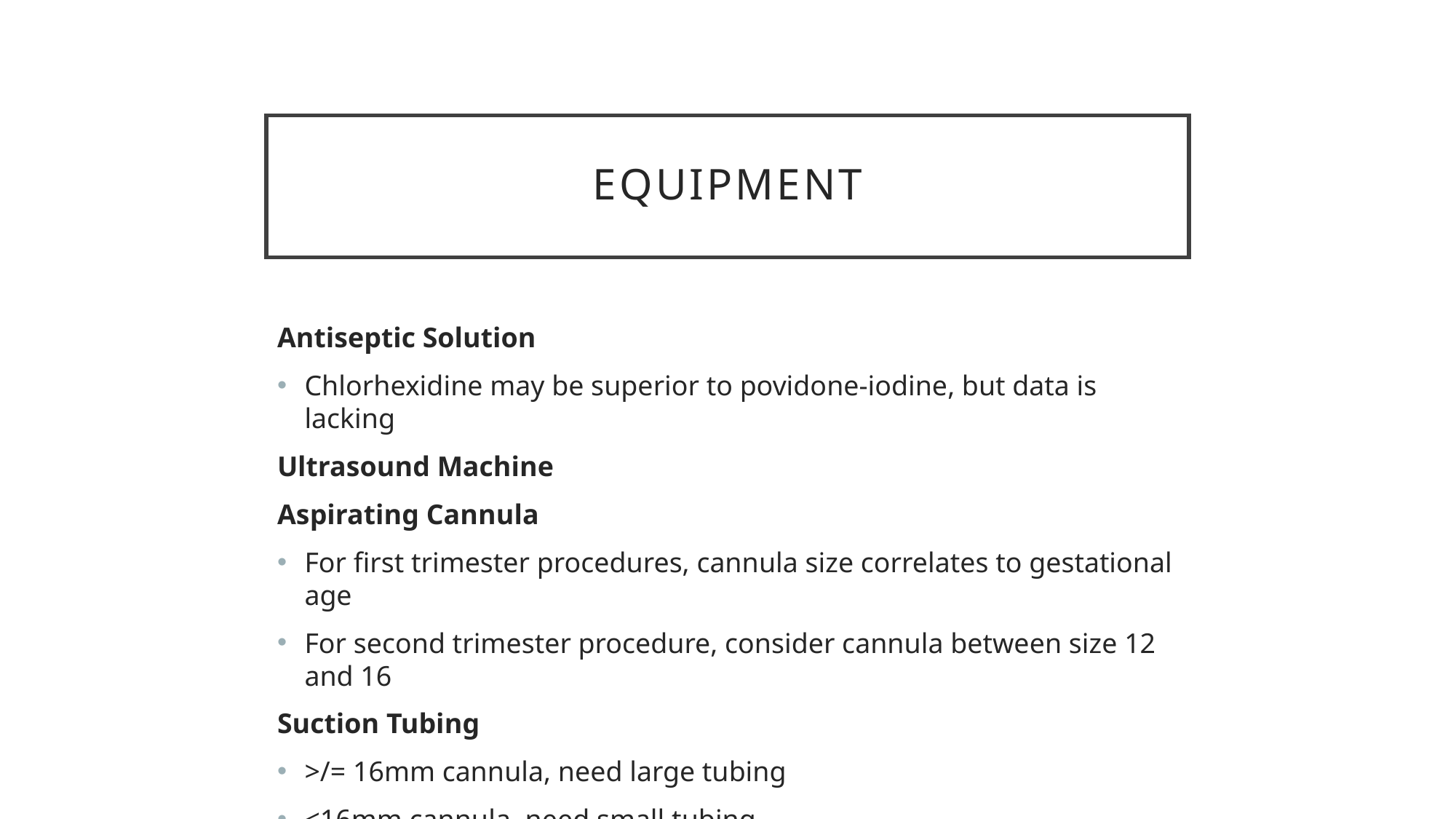

# Equipment
Antiseptic Solution
Chlorhexidine may be superior to povidone-iodine, but data is lacking
Ultrasound Machine
Aspirating Cannula
For first trimester procedures, cannula size correlates to gestational age
For second trimester procedure, consider cannula between size 12 and 16
Suction Tubing
>/= 16mm cannula, need large tubing
<16mm cannula, need small tubing

## Slide 9
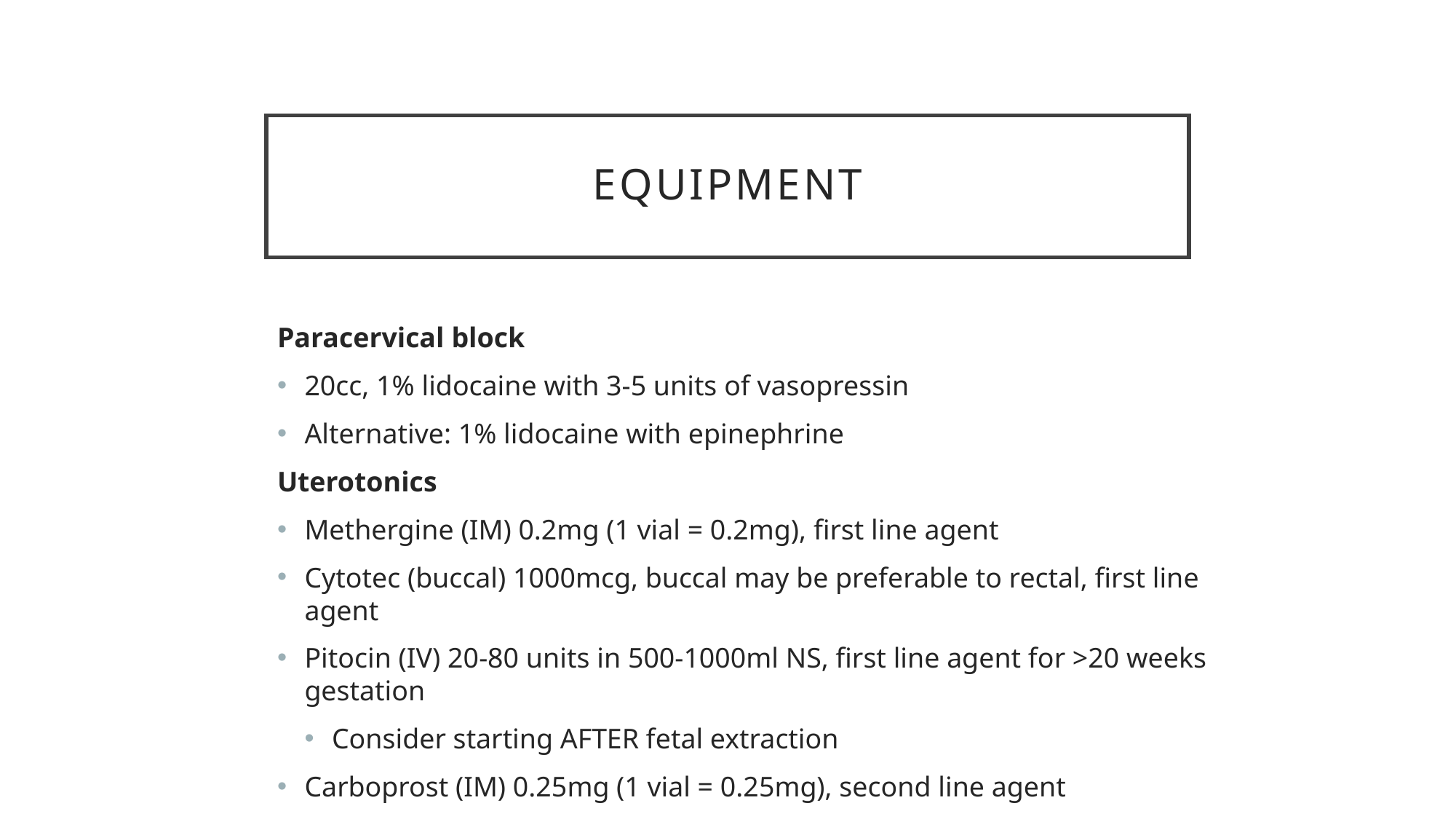

# EQUIPMENT
Paracervical block
20cc, 1% lidocaine with 3-5 units of vasopressin
Alternative: 1% lidocaine with epinephrine
Uterotonics
Methergine (IM) 0.2mg (1 vial = 0.2mg), first line agent
Cytotec (buccal) 1000mcg, buccal may be preferable to rectal, first line agent
Pitocin (IV) 20-80 units in 500-1000ml NS, first line agent for >20 weeks gestation
Consider starting AFTER fetal extraction
Carboprost (IM) 0.25mg (1 vial = 0.25mg), second line agent

## Slide 10
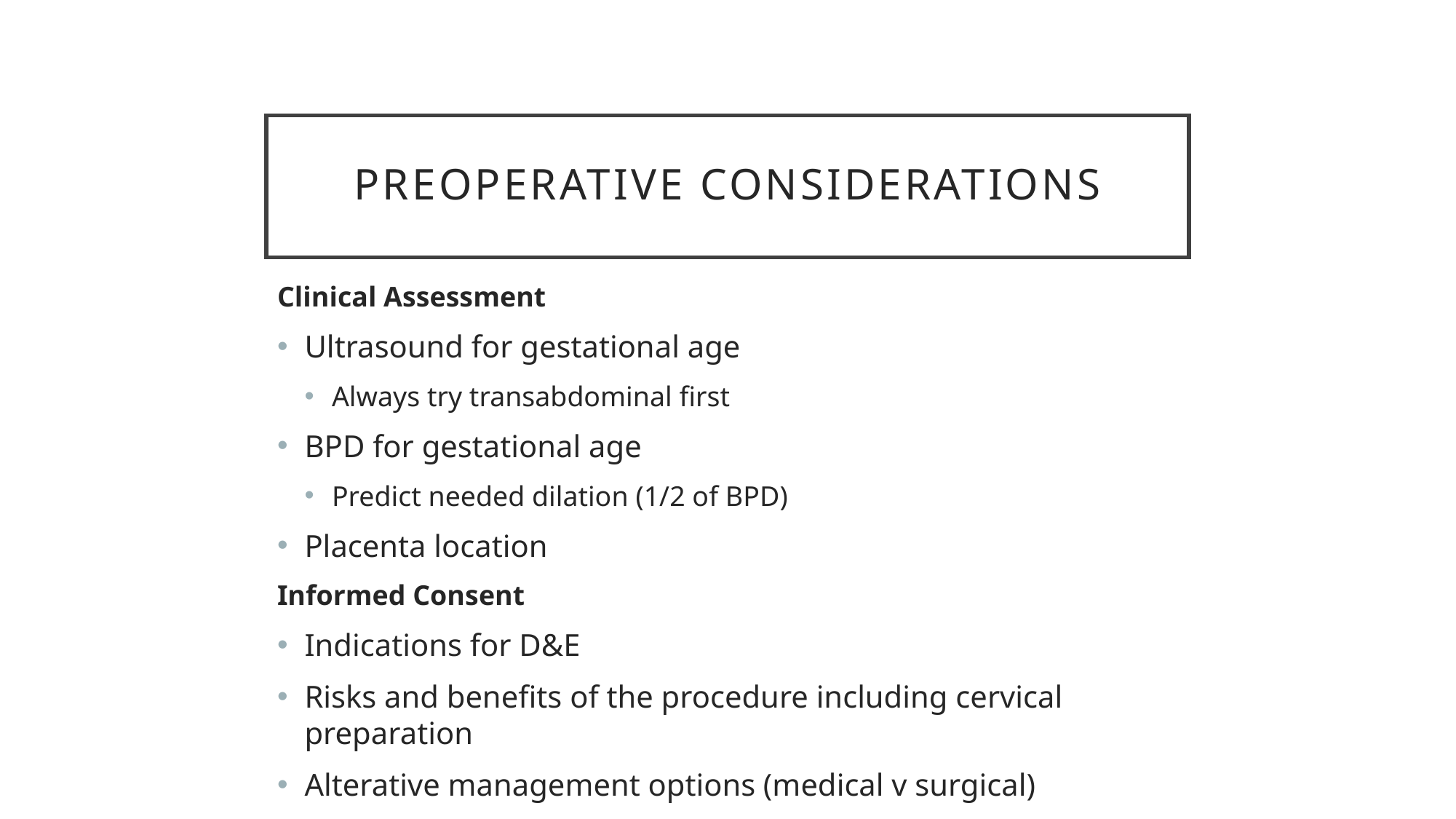

# Preoperative Considerations
Clinical Assessment
Ultrasound for gestational age
Always try transabdominal first
BPD for gestational age
Predict needed dilation (1/2 of BPD)
Placenta location
Informed Consent
Indications for D&E
Risks and benefits of the procedure including cervical preparation
Alterative management options (medical v surgical)
Alternative pregnancy outcomes

## Slide 11
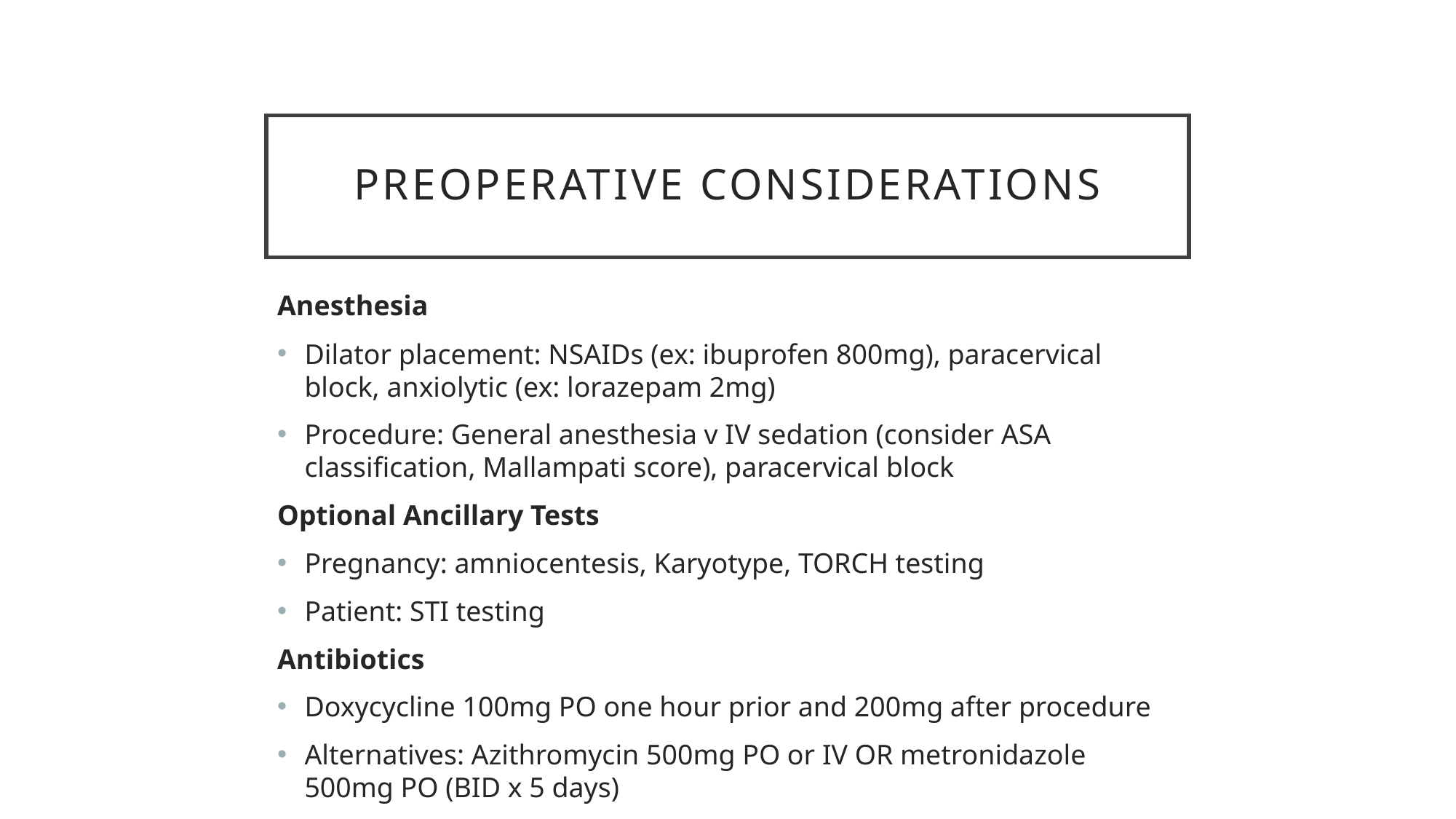

# Preoperative Considerations
Anesthesia
Dilator placement: NSAIDs (ex: ibuprofen 800mg), paracervical block, anxiolytic (ex: lorazepam 2mg)
Procedure: General anesthesia v IV sedation (consider ASA classification, Mallampati score), paracervical block
Optional Ancillary Tests
Pregnancy: amniocentesis, Karyotype, TORCH testing
Patient: STI testing
Antibiotics
Doxycycline 100mg PO one hour prior and 200mg after procedure
Alternatives: Azithromycin 500mg PO or IV OR metronidazole 500mg PO (BID x 5 days)

## Slide 12
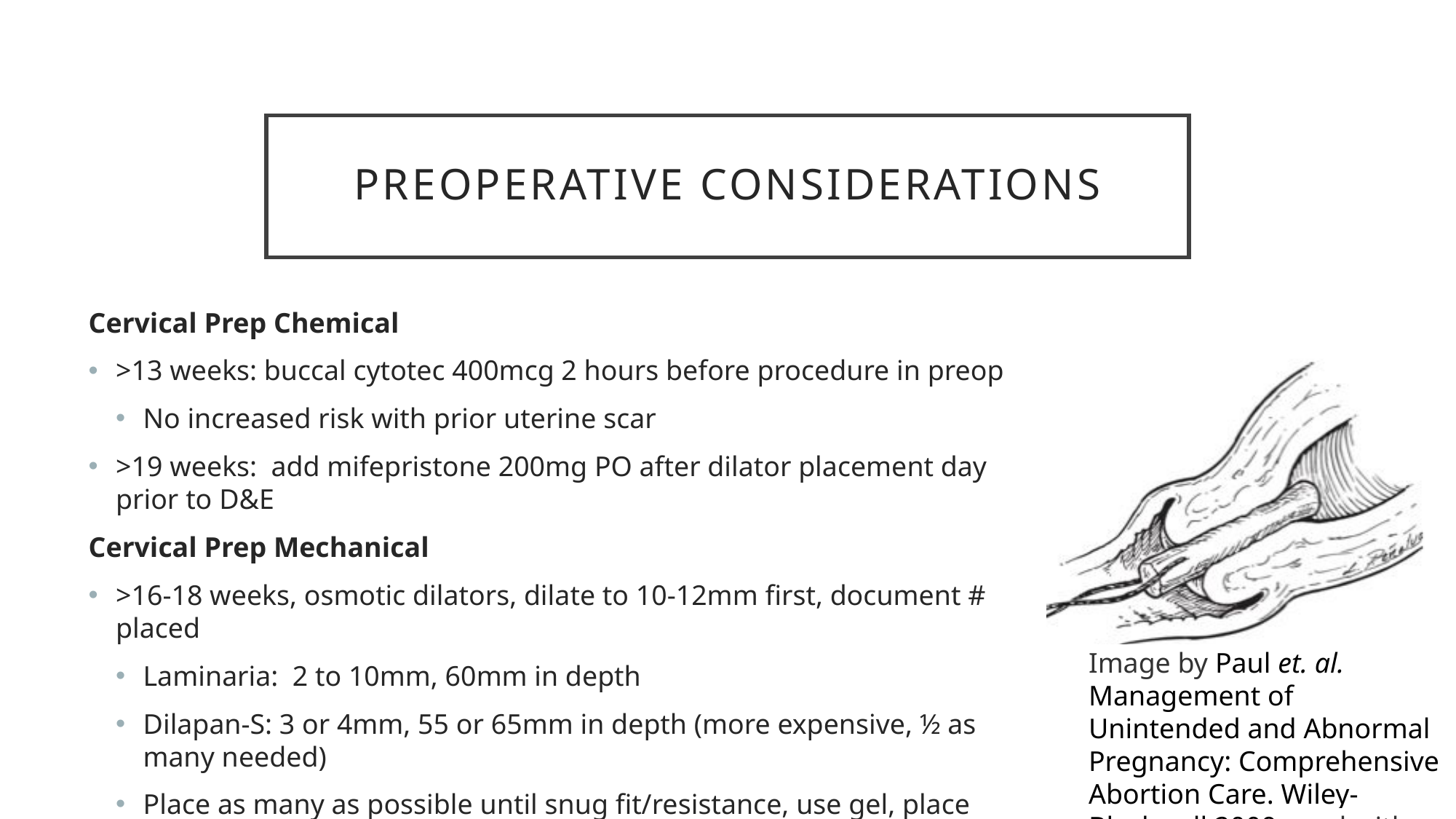

# Preoperative considerations
Cervical Prep Chemical
>13 weeks: buccal cytotec 400mcg 2 hours before procedure in preop
No increased risk with prior uterine scar
>19 weeks: add mifepristone 200mg PO after dilator placement day prior to D&E
Cervical Prep Mechanical
>16-18 weeks, osmotic dilators, dilate to 10-12mm first, document # placed
Laminaria: 2 to 10mm, 60mm in depth
Dilapan-S: 3 or 4mm, 55 or 65mm in depth (more expensive, ½ as many needed)
Place as many as possible until snug fit/resistance, use gel, place vaginal gauze
Bleeding after placement is normal if not heavier than a period and no need to change plan if membrane rupture with dilators but consider starting antibiotics
Image by Paul et. al. Management of Unintended and Abnormal Pregnancy: Comprehensive Abortion Care. Wiley-Blackwell 2009 used with permission.

## Slide 13
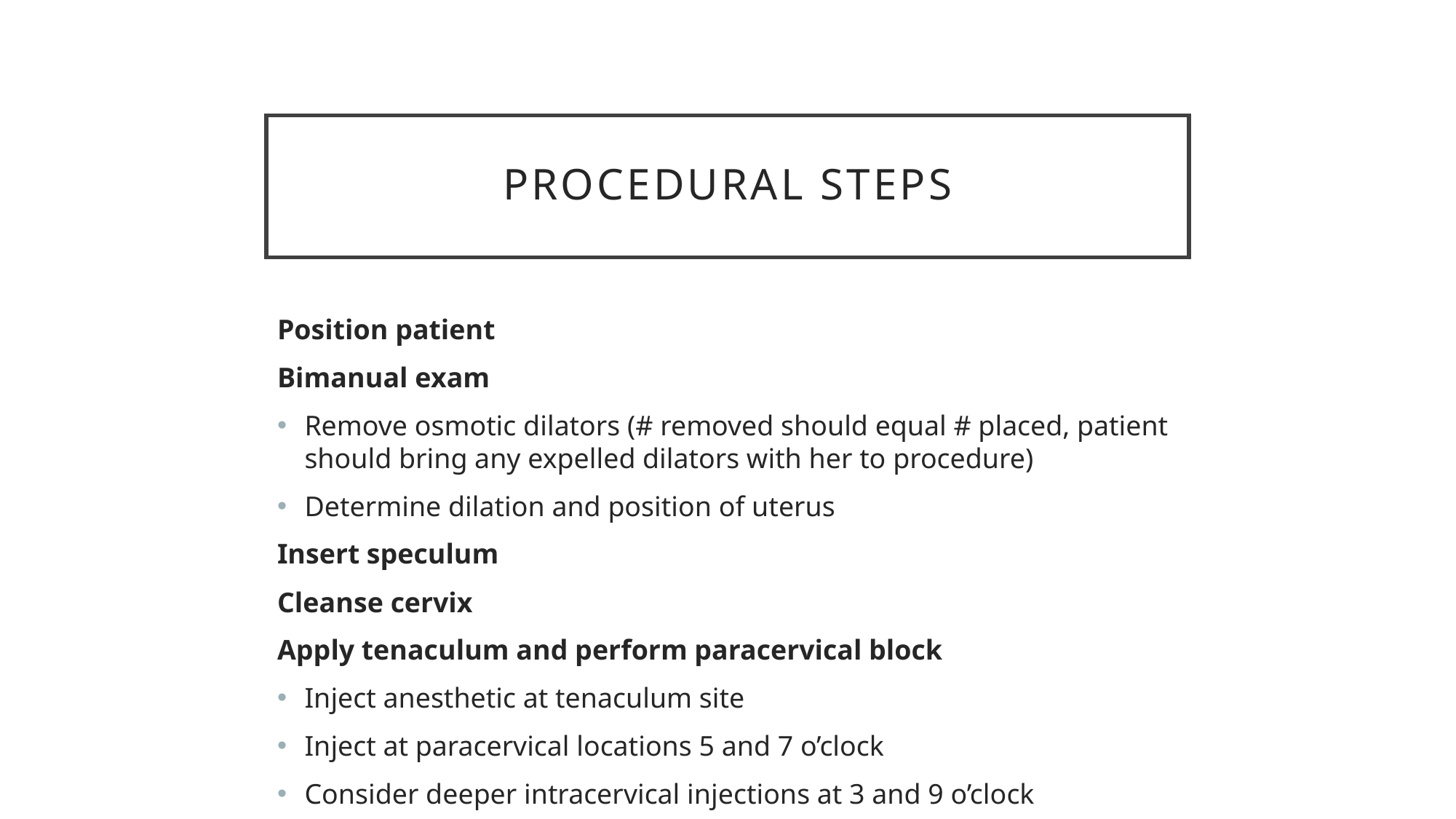

# Procedural Steps
Position patient
Bimanual exam
Remove osmotic dilators (# removed should equal # placed, patient should bring any expelled dilators with her to procedure)
Determine dilation and position of uterus
Insert speculum
Cleanse cervix
Apply tenaculum and perform paracervical block
Inject anesthetic at tenaculum site
Inject at paracervical locations 5 and 7 o’clock
Consider deeper intracervical injections at 3 and 9 o’clock

## Slide 14
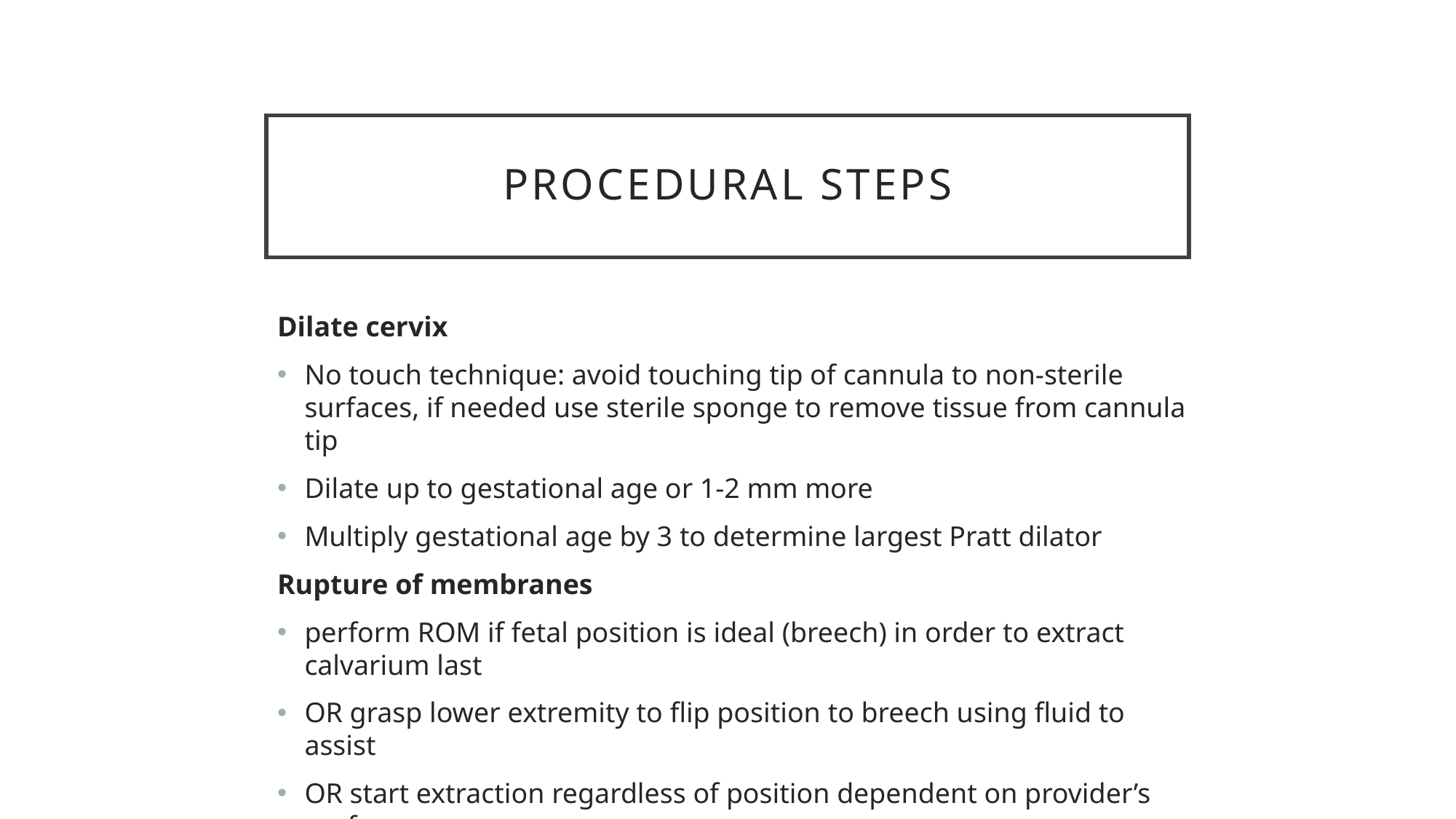

# PROCEDURAL STEPS
Dilate cervix
No touch technique: avoid touching tip of cannula to non-sterile surfaces, if needed use sterile sponge to remove tissue from cannula tip
Dilate up to gestational age or 1-2 mm more
Multiply gestational age by 3 to determine largest Pratt dilator
Rupture of membranes
perform ROM if fetal position is ideal (breech) in order to extract calvarium last
OR grasp lower extremity to flip position to breech using fluid to assist
OR start extraction regardless of position dependent on provider’s preference

## Slide 15
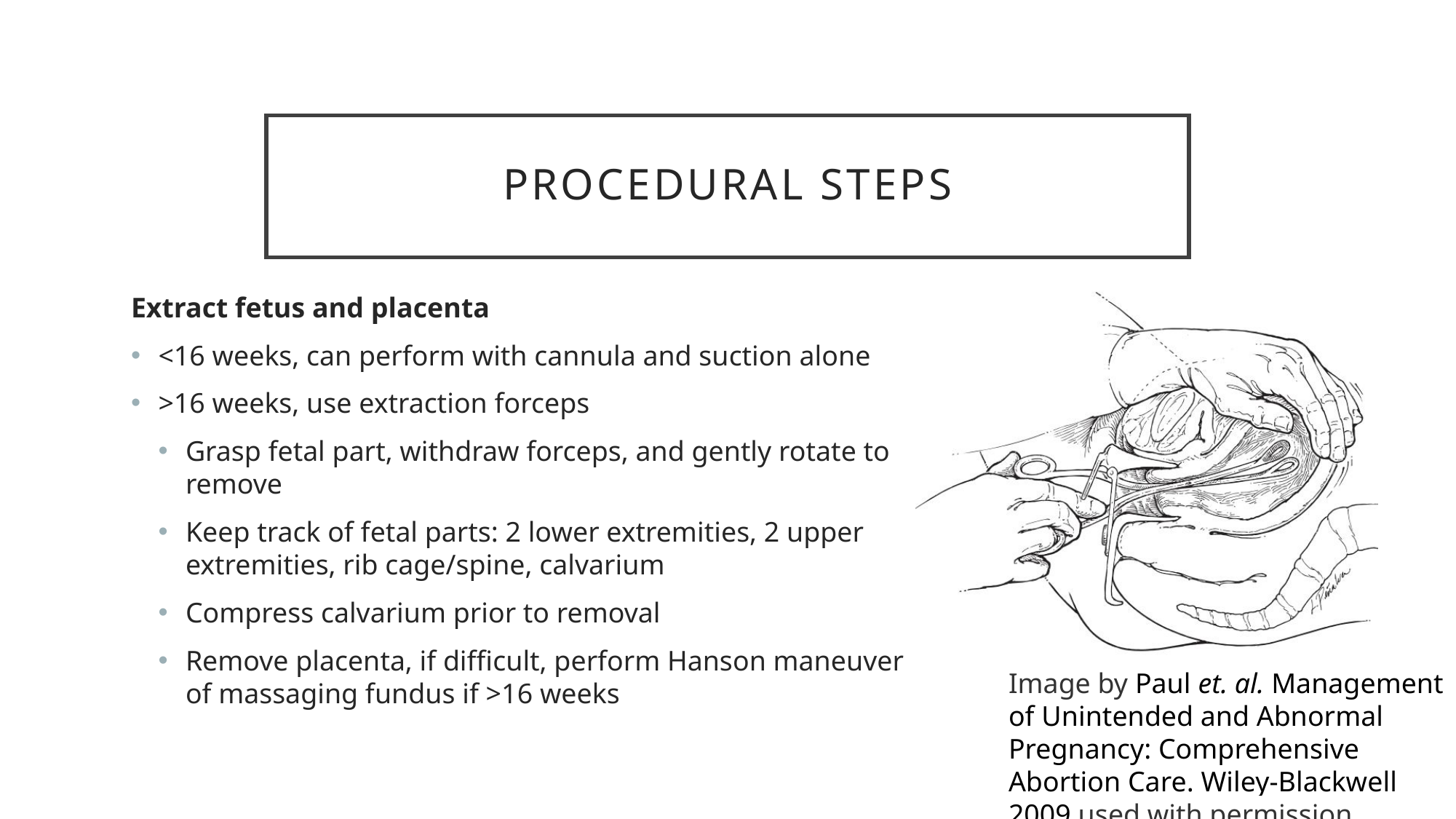

# PROCEDURAL STEPS
Extract fetus and placenta
<16 weeks, can perform with cannula and suction alone
>16 weeks, use extraction forceps
Grasp fetal part, withdraw forceps, and gently rotate to remove
Keep track of fetal parts: 2 lower extremities, 2 upper extremities, rib cage/spine, calvarium
Compress calvarium prior to removal
Remove placenta, if difficult, perform Hanson maneuver of massaging fundus if >16 weeks
Image by Paul et. al. Management of Unintended and Abnormal Pregnancy: Comprehensive Abortion Care. Wiley-Blackwell 2009 used with permission.

## Slide 16
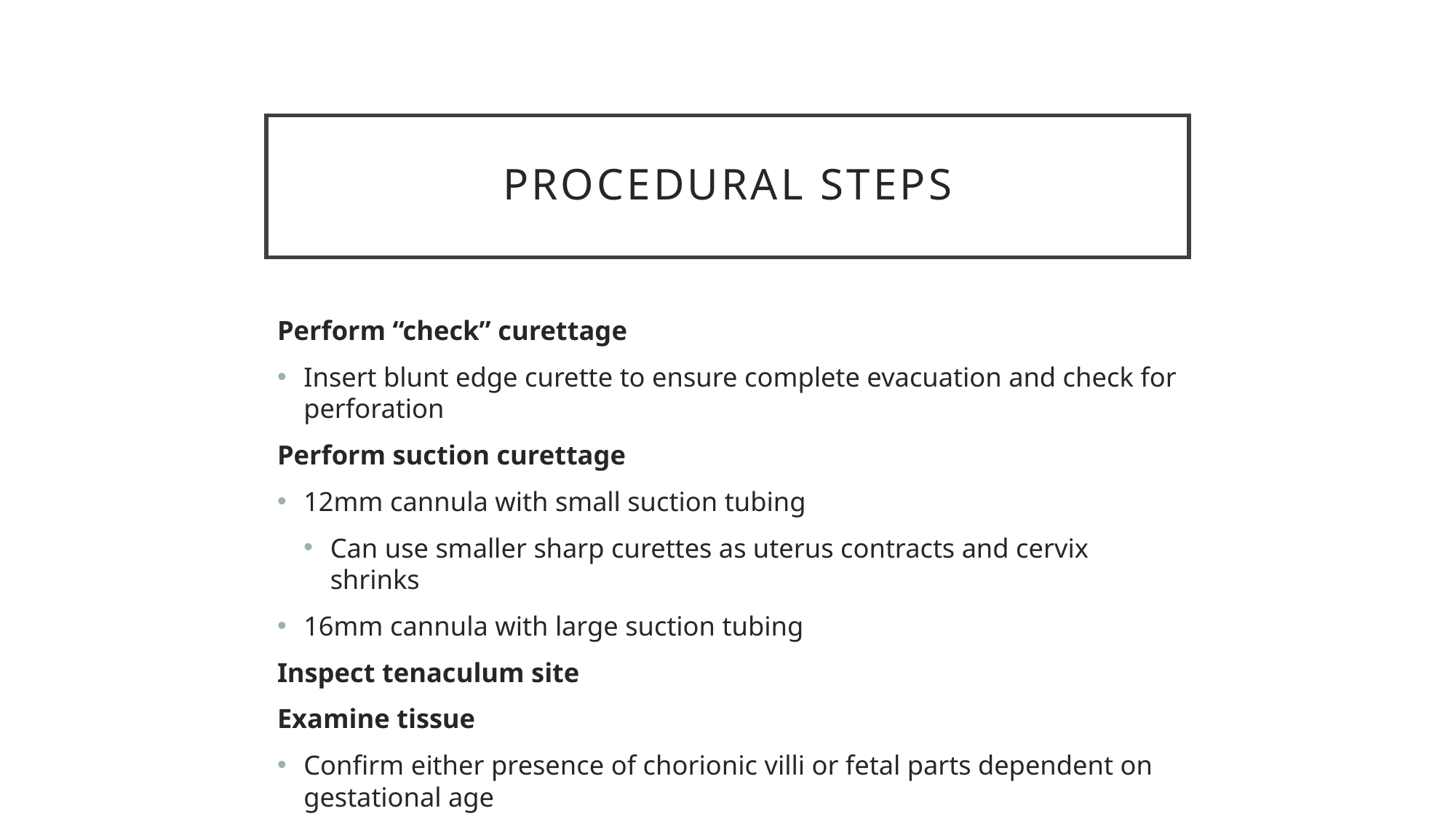

# PROCEDURAL STEPS
Perform “check” curettage
Insert blunt edge curette to ensure complete evacuation and check for perforation
Perform suction curettage
12mm cannula with small suction tubing
Can use smaller sharp curettes as uterus contracts and cervix shrinks
16mm cannula with large suction tubing
Inspect tenaculum site
Examine tissue
Confirm either presence of chorionic villi or fetal parts dependent on gestational age

## Slide 17
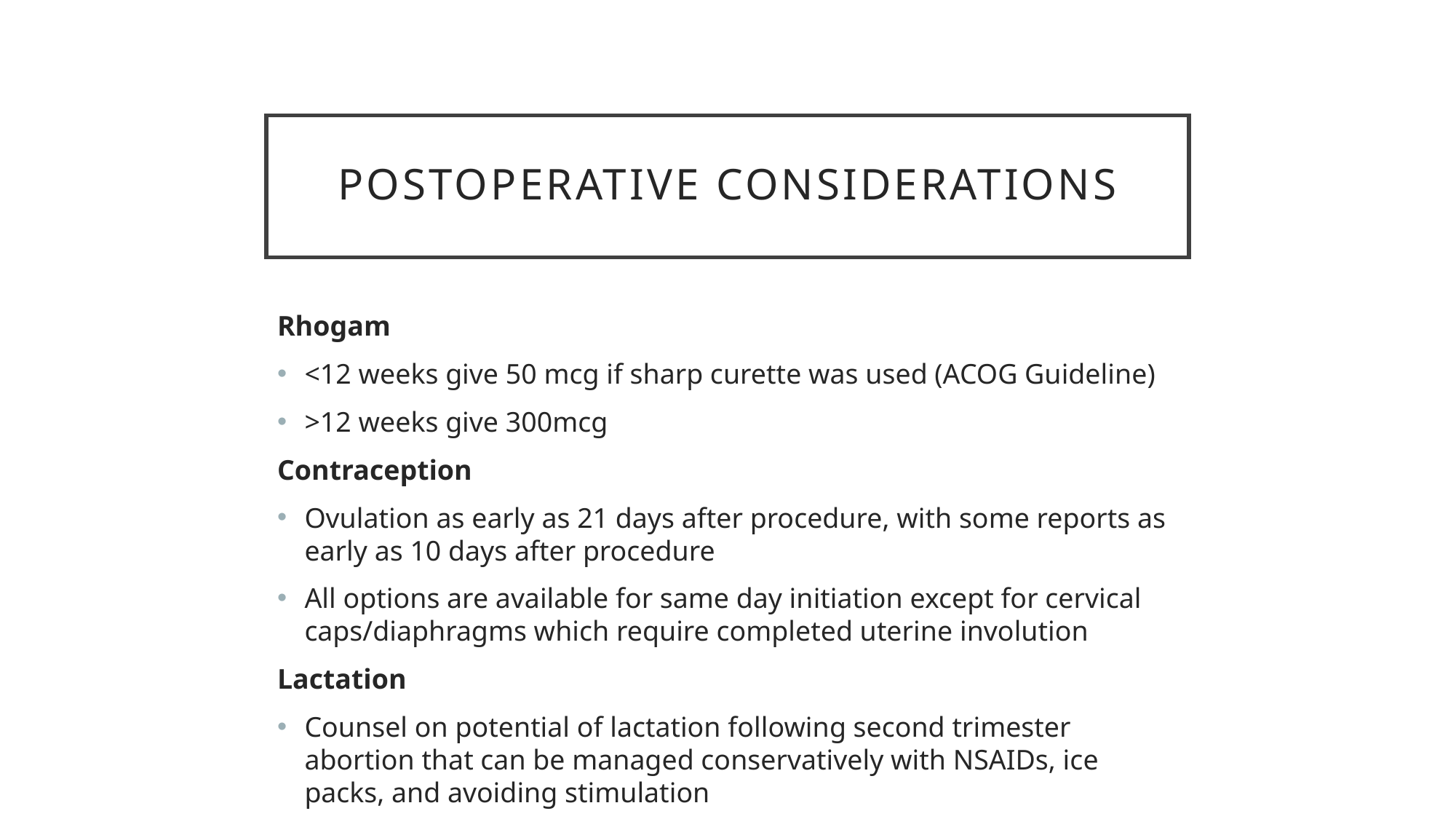

# Postoperative Considerations
Rhogam
<12 weeks give 50 mcg if sharp curette was used (ACOG Guideline)
>12 weeks give 300mcg
Contraception
Ovulation as early as 21 days after procedure, with some reports as early as 10 days after procedure
All options are available for same day initiation except for cervical caps/diaphragms which require completed uterine involution
Lactation
Counsel on potential of lactation following second trimester abortion that can be managed conservatively with NSAIDs, ice packs, and avoiding stimulation

## Slide 18
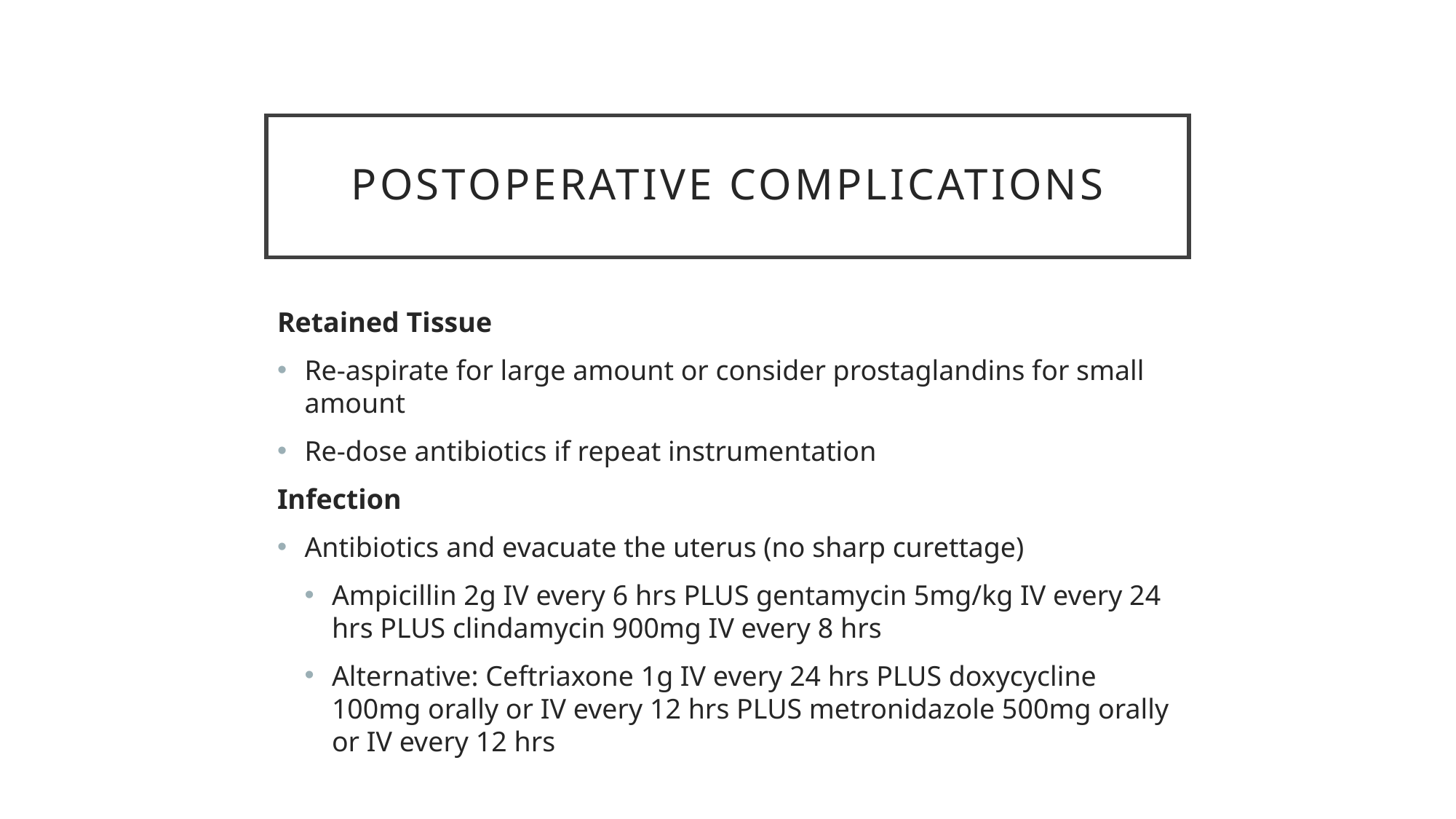

# Postoperative Complications
Retained Tissue
Re-aspirate for large amount or consider prostaglandins for small amount
Re-dose antibiotics if repeat instrumentation
Infection
Antibiotics and evacuate the uterus (no sharp curettage)
Ampicillin 2g IV every 6 hrs PLUS gentamycin 5mg/kg IV every 24 hrs PLUS clindamycin 900mg IV every 8 hrs
Alternative: Ceftriaxone 1g IV every 24 hrs PLUS doxycycline 100mg orally or IV every 12 hrs PLUS metronidazole 500mg orally or IV every 12 hrs

## Slide 19
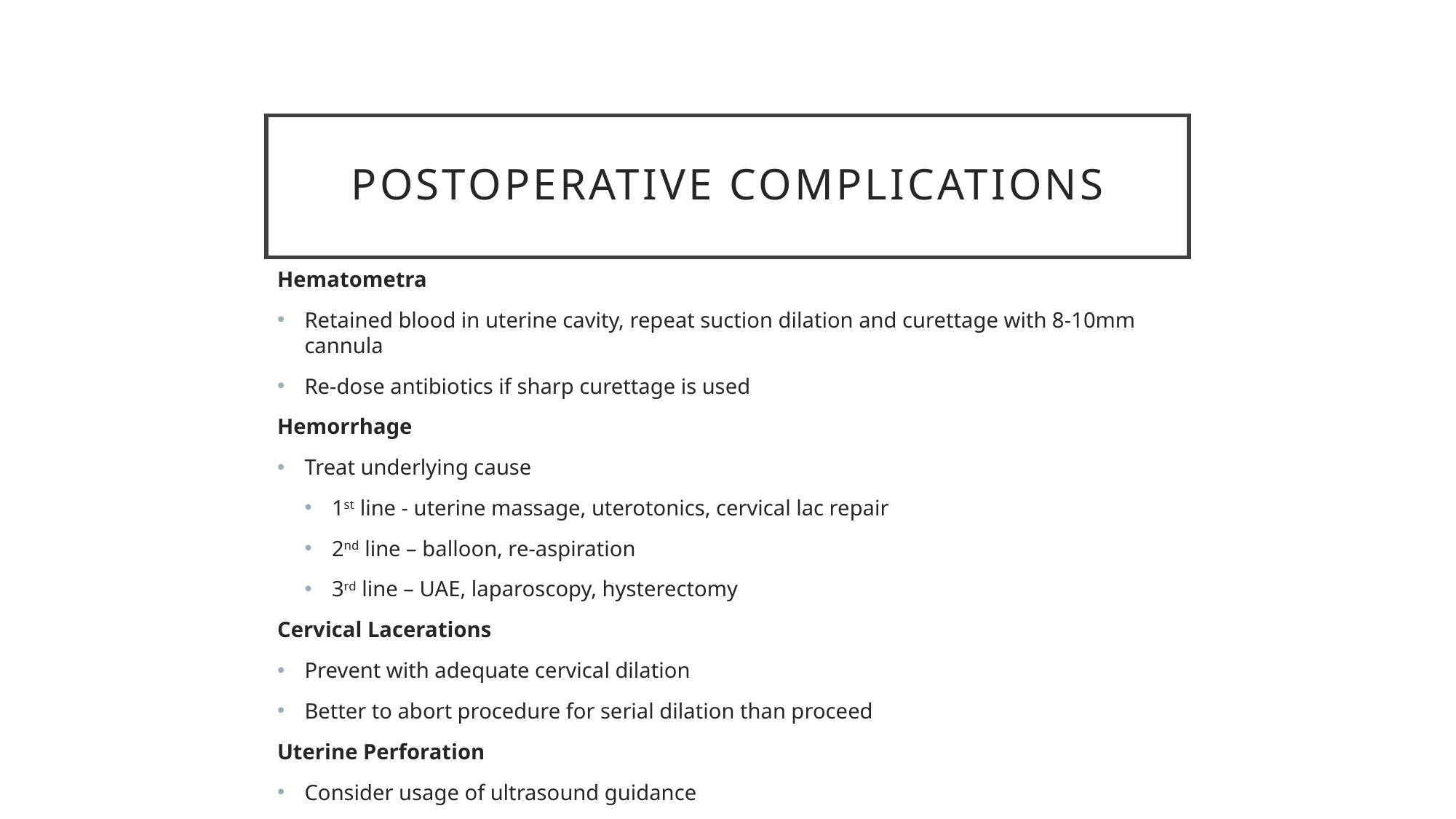

# Postoperative complications
Hematometra
Retained blood in uterine cavity, repeat suction dilation and curettage with 8-10mm cannula
Re-dose antibiotics if sharp curettage is used
Hemorrhage
Treat underlying cause
1st line - uterine massage, uterotonics, cervical lac repair
2nd line – balloon, re-aspiration
3rd line – UAE, laparoscopy, hysterectomy
Cervical Lacerations
Prevent with adequate cervical dilation
Better to abort procedure for serial dilation than proceed
Uterine Perforation
Consider usage of ultrasound guidance
Examine at end of procedure

## Slide 20
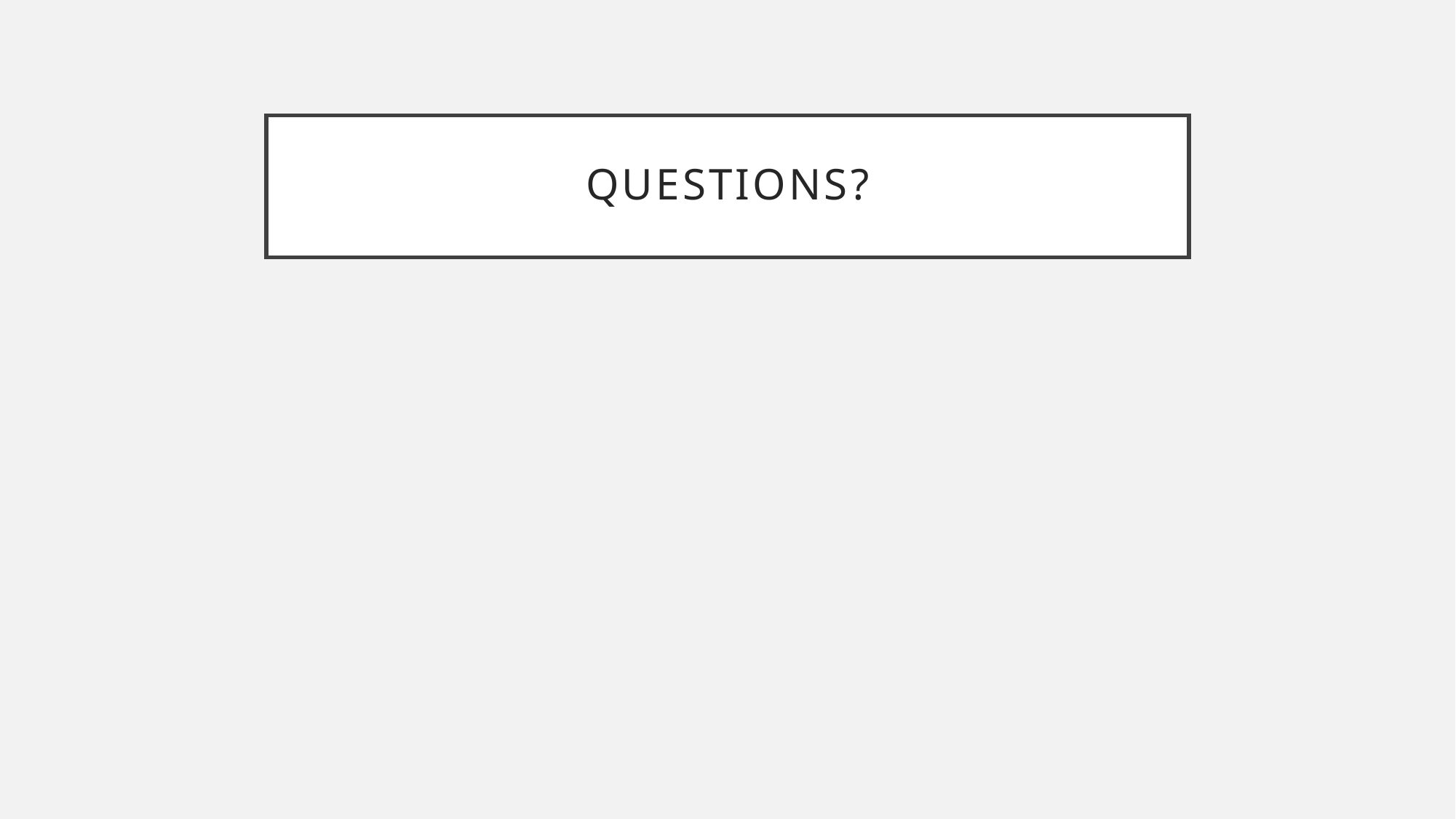

# Questions?

## Slide 21
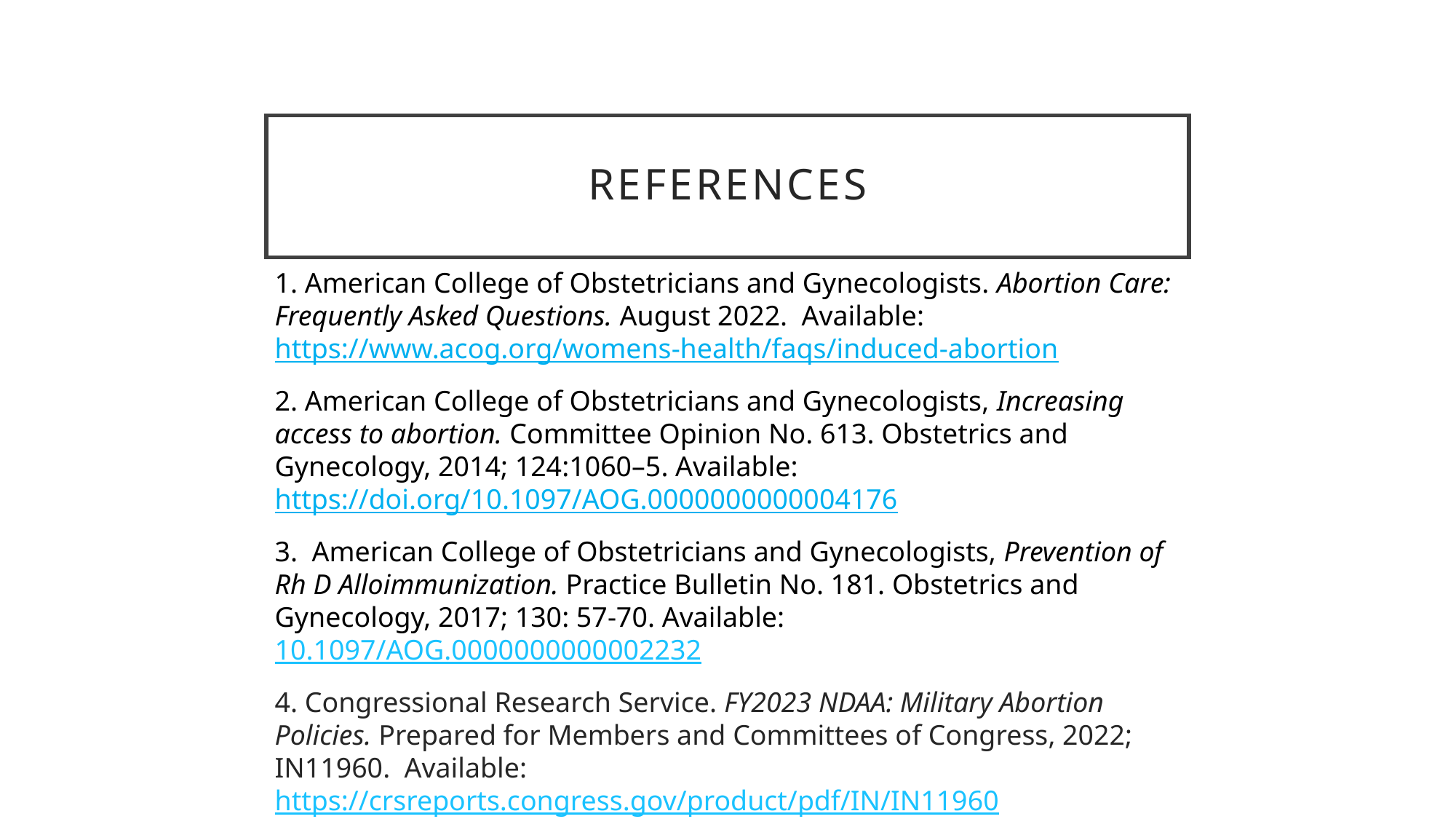

# References
1. American College of Obstetricians and Gynecologists. Abortion Care: Frequently Asked Questions. August 2022. Available: https://www.acog.org/womens-health/faqs/induced-abortion
2. American College of Obstetricians and Gynecologists, Increasing access to abortion. Committee Opinion No. 613. Obstetrics and Gynecology, 2014; 124:1060–5. Available: https://doi.org/10.1097/AOG.0000000000004176
3. American College of Obstetricians and Gynecologists, Prevention of Rh D Alloimmunization. Practice Bulletin No. 181. Obstetrics and Gynecology, 2017; 130: 57-70. Available: 10.1097/AOG.0000000000002232
4. Congressional Research Service. FY2023 NDAA: Military Abortion Policies. Prepared for Members and Committees of Congress, 2022; IN11960. Available: https://crsreports.congress.gov/product/pdf/IN/IN11960
5. Defense Department. Abortion Policy (1) of the Philip Buchen Files. Gerald R. Ford Presidential Library. Available: https://www.fordlibrarymuseum.gov/library/document/0019/4520504.pdf

## Slide 22
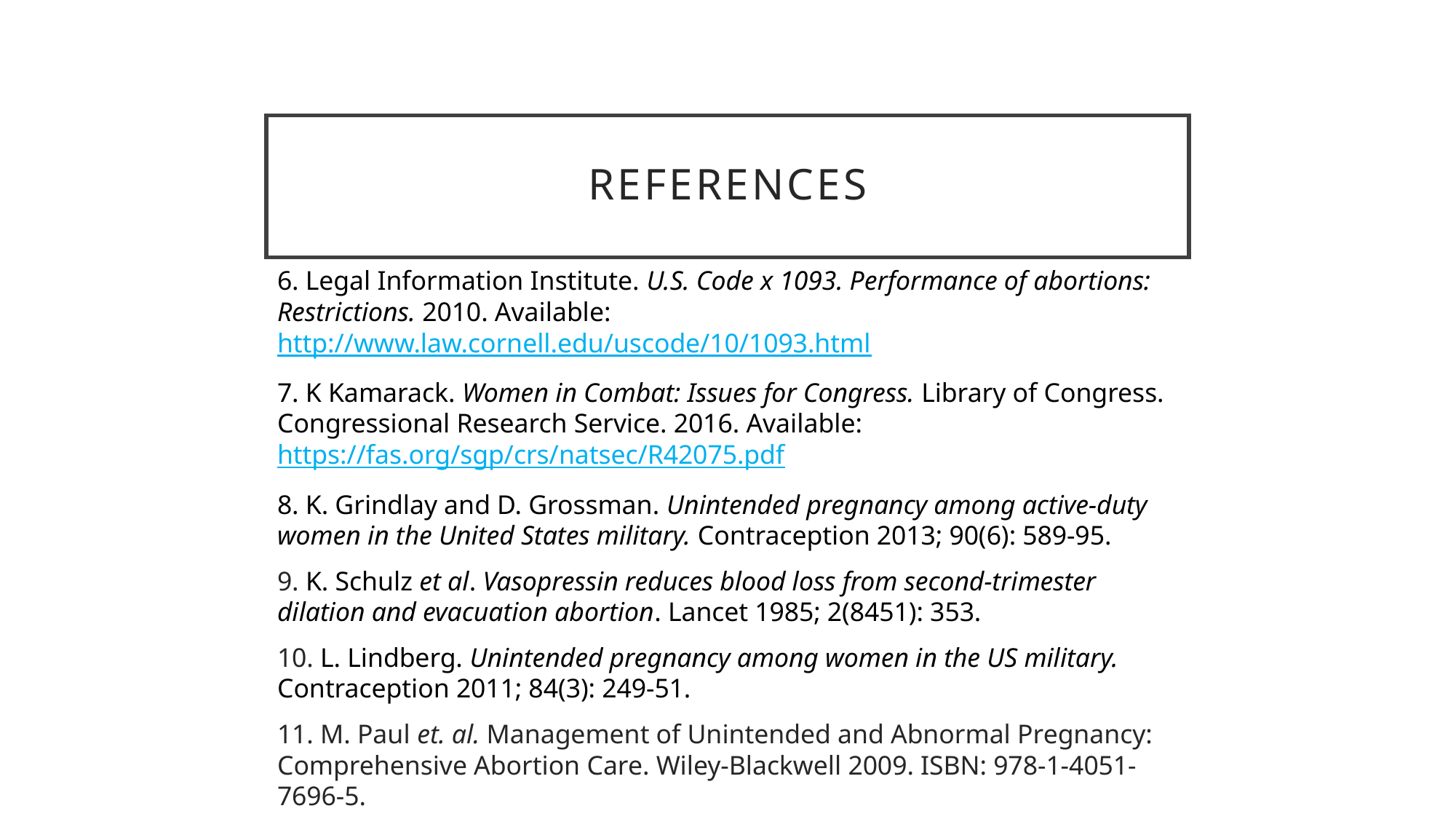

# References
6. Legal Information Institute. U.S. Code x 1093. Performance of abortions: Restrictions. 2010. Available: http://www.law.cornell.edu/uscode/10/1093.html
7. K Kamarack. Women in Combat: Issues for Congress. Library of Congress. Congressional Research Service. 2016. Available: https://fas.org/sgp/crs/natsec/R42075.pdf
8. K. Grindlay and D. Grossman. Unintended pregnancy among active-duty women in the United States military. Contraception 2013; 90(6): 589-95.
9. K. Schulz et al. Vasopressin reduces blood loss from second-trimester dilation and evacuation abortion. Lancet 1985; 2(8451): 353.
10. L. Lindberg. Unintended pregnancy among women in the US military. Contraception 2011; 84(3): 249-51.
11. M. Paul et. al. Management of Unintended and Abnormal Pregnancy: Comprehensive Abortion Care. Wiley-Blackwell 2009. ISBN: 978-1-4051-7696-5.

## Slide 23
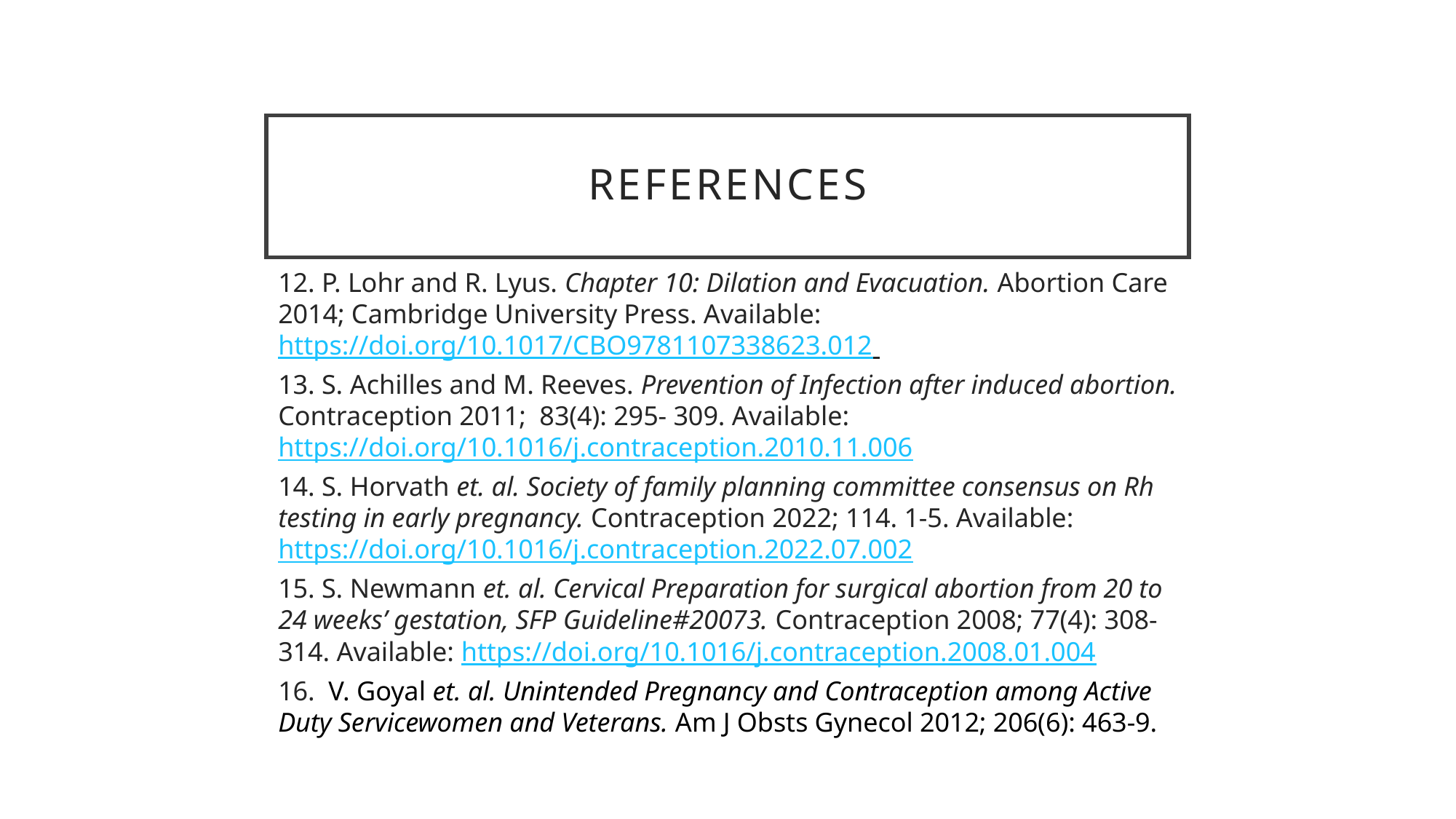

# References
12. P. Lohr and R. Lyus. Chapter 10: Dilation and Evacuation. Abortion Care 2014; Cambridge University Press. Available: https://doi.org/10.1017/CBO9781107338623.012
13. S. Achilles and M. Reeves. Prevention of Infection after induced abortion. Contraception 2011; 83(4): 295- 309. Available: https://doi.org/10.1016/j.contraception.2010.11.006
14. S. Horvath et. al. Society of family planning committee consensus on Rh testing in early pregnancy. Contraception 2022; 114. 1-5. Available: https://doi.org/10.1016/j.contraception.2022.07.002
15. S. Newmann et. al. Cervical Preparation for surgical abortion from 20 to 24 weeks’ gestation, SFP Guideline#20073. Contraception 2008; 77(4): 308-314. Available: https://doi.org/10.1016/j.contraception.2008.01.004
16. V. Goyal et. al. Unintended Pregnancy and Contraception among Active Duty Servicewomen and Veterans. Am J Obsts Gynecol 2012; 206(6): 463-9.

## Slide 24
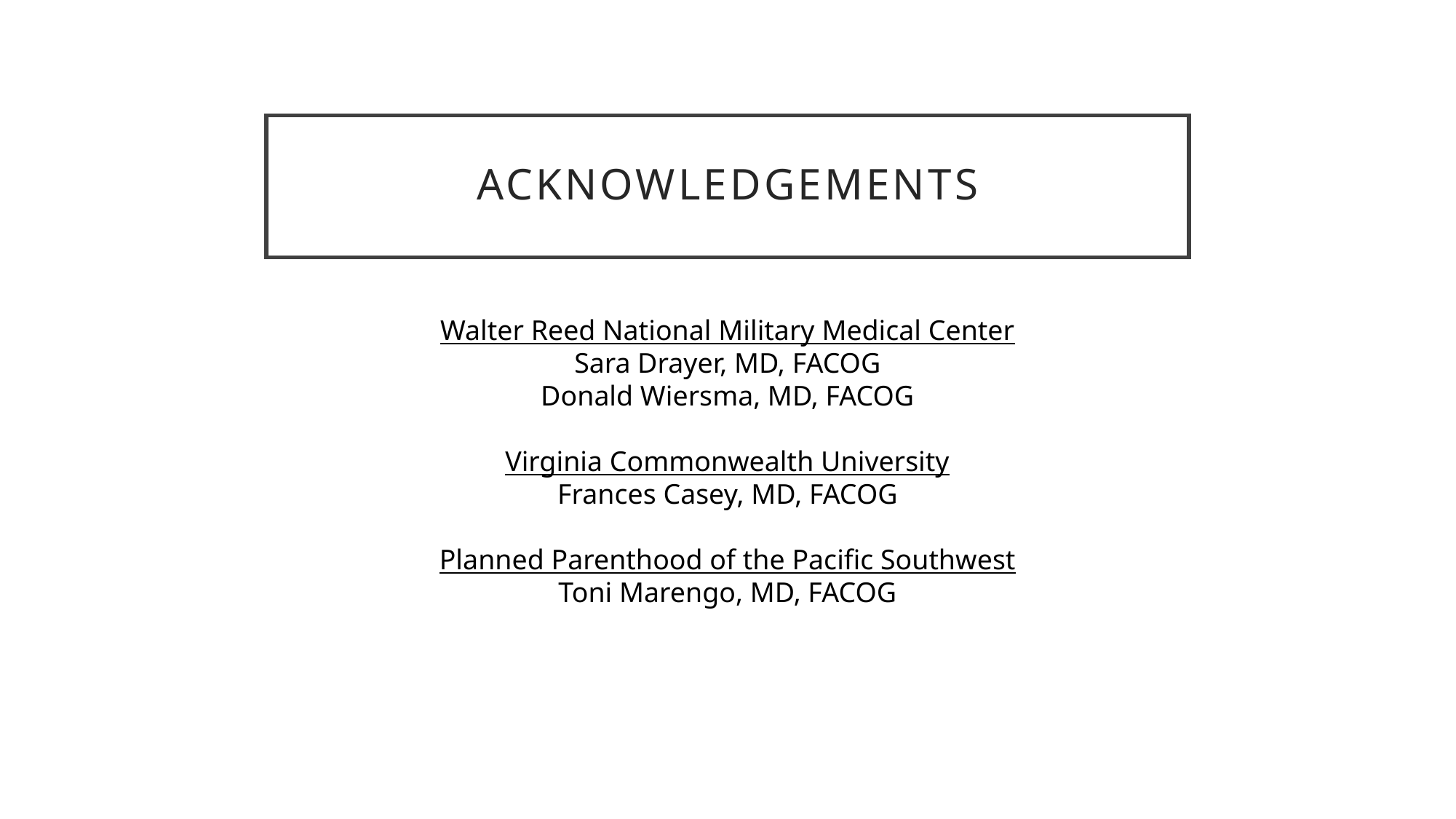

# Acknowledgements
Walter Reed National Military Medical Center
Sara Drayer, MD, FACOG
Donald Wiersma, MD, FACOG
Virginia Commonwealth University
Frances Casey, MD, FACOG
Planned Parenthood of the Pacific Southwest
Toni Marengo, MD, FACOG
