# Supplementary material for: Second Trimester Abortion: A Dilation and Evacuation Simulation for Gynecologic Surgery and Obstetrics Residents
Source: MedEdPORTAL. 2025 Jan 21;21:11489. doi: 10.15766/mep_2374-8265.11489 (PMC11753717; doi:10.15766/mep_2374-8265.11489)
Supplement: Supplementary file 1 — Materials and Instructions.docxFacilitator Guide.docxLearner Grading Rubric.docxSimulation Debrief.pptxSpeaker Notes for Debrief.docxPre- and Postsimulation Assessment.docxSimulation Video.movFacilitator Sequence of Events.docx [file mep_2374-8265.11489-s001.zip › E. Speaker Notes for Debrief.docx]

Appendix E: Facilitator Script for Debrief PowerPoint

*Purpose is to review with learners a standard dilation and evacuation checklist as well as preoperative and post-operative considerations, total time: 10 minutes*

**Slide 1:** Introduction to the Debrief

**Slide 2:** Disclaimer

**Slide 3:** Review Objectives

**Slide 4:** Discussion and reflection on the simulation (approximately 5 min)

**Slide 5:** A dilation and evacuation (D&E) is the safest option for patients undergoing a second trimester abortion as D&Es have an overall lower complication rate compared to medical management. A seminal randomized control trial (RCT) from the UK showed that women who were randomized to a D&E were more likely to choose the same method in the future compared to those randomized to an induction of labor (Grimes, 2004). One large RCT in the US had to be stopped early as too many women declined randomization and preferred a D&E (Kelly, 2010). The American College of Obstetricians and Gynecologists (ACOG) recommends that residency programs offer hands-on training in second trimester D&Es (Practice Bulletin No. 162). Military residents must opt into this type of training, and often rely on civilian residency programs for the needed exposure. However, the future of training in dilation and evacuations is uncertain as a recent study published in August 2022 in the green journal demonstrated that 128 of the 268 current ACGME OB/GYN resident programs are in states certain or likely to ban abortion in the post-Roe world (Vinekar, 2022).

**Slide 6:** We queried the primary sources that drive the Society of Family Planning (SFP) guidelines, ACOG recommendations, and national the Ryan curriculum to create an evidence-based protocol. We then asked expert reviewers to adjust the protocol for clinical practice as needed.

**Slide 7:** The protocol starts by listing the necessary equipment to include instruments for a speculum, tenaculum, dilators, and extraction forceps. We detailed optimal situations for each type of forceps depending on the clinical circumstance.

**Slide 8:** We then discuss the type of antiseptic solution, aspiration cannula, and suction tubing. We do also include consideration of an ultrasound, though no data supports that ultrasounds directly enhance the safety of this procedure unless in the context of a teaching hospital.

**Slide 9:** Lastly for equipment, the protocol details a paracervical block and first line uterotonics. Family planning literature advocates the addition of vasopressin to reduce blood loss which has been supported in double blinded randomized trials in patients undergoing D&Es (Schultz 1985). There is limited efficacy to support routine prophylactic use of uterotonics, though a recent retrospective analysis published in this month’s green journal found an association between prophylactic medication use and lower operative hemorrhage rate (21% v 57%) (Fairchild, 2022). Most experts advocate for prophylactic use of uterotonics in patients undergoing a second trimester D&Es with risk factors for uterine atony such as intrauterine infection and general anesthesia using halogenated gasses. The protocol lists the standard first line and second line agents and route of administration.

**Slide 10:** We then listed preoperative considerations including measurements of gestational age,

specifically the biparietal diameter to predict the needed cervical dilation for the procedure as well as important aspects of informed consent.

**Slide 11:** The protocol also lists ways to distinguish if patients are candidates for IV sedation v general anesthesia based on comorbidities as well as a list of optional ancillary tests especially in the context of an IUFD. Lastly antibiotics are recommended by all major societies, and the protocol references ACOG’s recommendations for primary agents (Practice Bulletin No. 135) and the SFP recommendations (Guideline 20102) for alternative agents.

**Slide 12:** The last and arguably most important preoperative consideration is cervical preparation. The

protocol details both chemical and mechanical agents with recommendations based on standard protocols in the literature (Paul, 2009) as well as protocols practiced by surrounding civilian institutions. A photo is included to illustrate proper placement of osmotic dilators. Image by Paul *et. al.* Management of Unintended and Abnormal Pregnancy: Comprehensive Abortion Care. Wiley-Blackwell 2009 used with permission.

**Slide 13:** The protocol then details the procedural steps. For the paracervical block, some experts advocate for standard injection at the vaginal reflection at 5 and 7 o'clock while others argue for a deep intracervical injection at 3 and 9 o’clock. Neither method has been demonstrated to be superior.

**Slide 14:** Tips to dilate the cervix are included as well as an emphasis on a no touch sterile technique that comes from guidance for manual aspiration in the first trimester. The timing of membrane rupture is also discussed.

**Slide 15:** Techniques to extract the fetus and placenta are then explained, with an illustration of maneuvers one can employ in cases of an adherent placenta.

**Slide 16:** Lastly, the protocol discusses the final curettage as well examining the tissue to confirm complete evacuation. There is a depiction of the Hason maneuver for those less familiar with the technique. Image by Paul *et. al.* Management of Unintended and Abnormal Pregnancy: Comprehensive Abortion Care. Wiley-Blackwell 2009 used with permission.

**Slide 17:** For postoperative considerations, the protocol aligns with ACOG’s recommendation (Practice

Bulletin No. 181) of giving 300 mcg dose of RhoGAM if the gestational age was greater than 12 weeks. Data published by the Society of Family Planning now suggests that giving RhoGAM before 12 weeks is only necessary if sharp curette was used (Horvath 2022). The protocol then helps providers counsel patients on appropriate contraception and possibility of lactation following D&Es.

**Slide 18:** For standard complications, the protocol helps providers carry out appropriate treatments. For a post abortion infection, the protocol aligns with the CDC recommendations for antibiotic use (same regimen used for broad coverage postpartum endometritis for first line agents) and same regiment used for pelvic inflammatory disease for second line agents).

**Slide 19:** The less common complications are also discussed as well as strategies to prevent them.

**Slide 20:** What questions do you have?

**Slide 21 -23:** References

**Slide 24:** Acknowledgements
