# Supplementary material for: Second Trimester Abortion: A Dilation and Evacuation Simulation for Gynecologic Surgery and Obstetrics Residents
Source: MedEdPORTAL. 2025 Jan 21;21:11489. doi: 10.15766/mep_2374-8265.11489 (PMC11753717; doi:10.15766/mep_2374-8265.11489)
Supplement: Supplementary file 1 — Materials and Instructions.docxFacilitator Guide.docxLearner Grading Rubric.docxSimulation Debrief.pptxSpeaker Notes for Debrief.docxPre- and Postsimulation Assessment.docxSimulation Video.movFacilitator Sequence of Events.docx [file mep_2374-8265.11489-s001.zip › F. Pre- and Postsimulation Assessment.docx]

**Dilation & Evacuation: Pre- Simulation Training Questionnaire**

*Designed for learners prior to completion of the simulation, total time 2-3 minutes*

Do you consent to participate in this project?

- Yes
- No

What is your current level of training?

- Medical Student
- PGY-1
- PGY-2
- PGY-3
- PGY-4
- PGY-5
- PGY-6
- PGY-7
- Attending

I feel comfortable with my current level of knowledge, experience, and training regarding D&E (Dilation & Evacuation).

- Strongly Disagree
- Disagree
- Neutral
- Agree
- Strongly Agree

I know the steps of a D&E procedure.

- Strongly Disagree
- Disagree
- Neutral
- Agree
- Strongly Agree

Have you completed a family planning rotation or another rotation focused on teaching dilation and evacuation?

- Yes
- No

How many D&Es have you performed?

- None
- >5
- 5-10
- 10-20
- >20

Do you intend to offer D&E procedures in your future practice?

- Yes
- No

**Before** the simulation session, I am confident in performing a D&E (Dilation & Evacuation).

- Strongly Disagree
- Disagree
- Neutral
- Agree
- Strongly Agree

*Future Assessments should consider including the following question:*

I feel confident addressing the complications of a D&E procedure

- Strongly Disagree
- Disagree
- Neutral
- Agree
- Strongly Agree

**Dilation & Evacuation: Post- Simulation Training Questionnaire**

*Designed for learners after completion of the simulation, total time 5 minutes*

The simulation’s sequence and flow were appropriate.

- Strongly disagree
- Disagree
- Neutral
- Agree
- Strongly agree

The **overall simulation** was realistic compared to prior D&E simulations I have performed.

- Strongly disagree
- Disagree
- Neutral
- Agree
- Strongly agree
- N/A - I’ve never performed a D&E simulation before

The following components of the simulation were realistic compared to previous D&E procedures I have performed (please select one):

|  | **Overall Simulation** | **Fetal Model** | **Calvarium Model** | **Uterine Model** | **Cervical Model** | **Tactile Sensation** |
| --- | --- | --- | --- | --- | --- | --- |
| **Strongly Disagree** |  |  |  |  |  |  |
| **Disagree** |  |  |  |  |  |  |
| **Neutral** |  |  |  |  |  |  |
| **Agree** |  |  |  |  |  |  |
| **Strongly Agree** |  |  |  |  |  |  |
| **N/A - never performed D&E** |  |  |  |  |  |  |

**After** the simulation session, I am confident in performing a D&E.

- Strongly Disagree
- Disagree
- Neutral
- Agree
- Strongly Agree

**After the simulation**, I know the steps of a D&E procedure.

- Strongly Disagree
- Disagree
- Neutral
- Agree
- Strongly Agree

I feel I need additional training and preparation before performing a D&E.

- Strongly Disagree
- Disagree
- Neutral
- Agree
- Strongly Agree.

I feel that this simulation enhanced my understanding of a D&E.

- Strongly Disagree
- Disagree
- Neutral
- Agree
- Strongly Agree.

Do you intend to offer D&E procedures in your future practice?

- Yes
- No

How would you improve the simulation?

- Comments:

Any additional feedback?

- Comments:

*Future assessments should consider including the following question:*

After the simulation, I feel confident addressing the complications of a D&E procedure.

- Strongly Disagree
- Disagree
- Neutral
- Agree
- Strongly Agree
