# Supplementary material for: Second Trimester Abortion: A Dilation and Evacuation Simulation for Gynecologic Surgery and Obstetrics Residents
Source: MedEdPORTAL. 2025 Jan 21;21:11489. doi: 10.15766/mep_2374-8265.11489 (PMC11753717; doi:10.15766/mep_2374-8265.11489)
Supplement: Supplementary file 1 — Materials and Instructions.docxFacilitator Guide.docxLearner Grading Rubric.docxSimulation Debrief.pptxSpeaker Notes for Debrief.docxPre- and Postsimulation Assessment.docxSimulation Video.movFacilitator Sequence of Events.docx [file mep_2374-8265.11489-s001.zip › H. Facilitator Sequence of Events.docx]

**Facilitator’s Simulation Sequence of Events Guide**

*Designed for facilitators to review before and during the simulation for ease of use of different learning materials*

| **Activity** | **Required Participants** | **Activity Duration** | **Correlating Appendix** |
| --- | --- | --- | --- |
| 1. Pre-Simulation Assessment *(Optional)* | Facilitator  All Learners | 1 minute | Appendix F |
| 1. Instructional Video | Facilitator  All Learners | 7 minutes | Appendix G |
| 1. Preoperative Considerations Discussion | Facilitator  All Learners | 5 minutes | Appendix B |
| 1. Simulation: Preprocedural Steps | Facilitator  2 Learners | 4 minutes  *(2 minutes per learner)* | Appendix B |
| 1. Simulation: D&E | Facilitator  2 Learners | 10-20 minutes  *(5-10 minutes per learner)* | Appendix A  Appendix B |
| 1. Simulation: Postprocedural Steps | Facilitator  2 Learners | 4 minutes  *(2 minutes per learner)* | Appendix B |
| 1. Postoperative Considerations & Complications Discussion | Facilitator  All Learners | 5 minutes | Appendix B |
| 1. Facilitator Assessment of Learner | Facilitator | Completed during simulation | Appendix C |
| 1. Debrief | Facilitator  All Learners | 10 minutes | Appendix D  Appendix E |
| 1. Post-Simulation Assessment *(Optional)* | Facilitator  All Learners | 1 minute | Appendix F |
