# Supplementary material for: Second Trimester Abortion: A Dilation and Evacuation Simulation for Gynecologic Surgery and Obstetrics Residents
Source: MedEdPORTAL. 2025 Jan 21;21:11489. doi: 10.15766/mep_2374-8265.11489 (PMC11753717; doi:10.15766/mep_2374-8265.11489)
Supplement: Supplementary file 1 — Materials and Instructions.docxFacilitator Guide.docxLearner Grading Rubric.docxSimulation Debrief.pptxSpeaker Notes for Debrief.docxPre- and Postsimulation Assessment.docxSimulation Video.movFacilitator Sequence of Events.docx [file mep_2374-8265.11489-s001.zip › Educational Summary Report_11489.pdf]

MedEdPORTAL® The AAMC Journal of Teaching and Learning Resources

Original Publication

Open Access

Second Trimester Abortion: A Dilation and Evacuation Simulation for Gynecologic Surgery and Obstetrics Residents

Kiley Hunkler, MD, MSt, MSc*, David Boedeker, DO, MHA, Elizabeth Gill, MD, Katelyn Simpkins, MD, Katerina Shvartsman, MD, Jill Brown, MD, MPH, MHS, Sara Drayer, MD

*Corresponding author: Kiley.Hunkler@gmail.com

Abstract

Introduction: The future of training in second trimester surgical abortions with dilation and evacuation (D&E) procedures faces ongoing legal and political scrutiny; thus, adjuncts to standard clinical experiences are exceedingly important. We sought to build medical trainees' surgical familiarity with D&Es using a realistic simulation model. **Methods:** The simulation began with an instructional video reviewing accessible and affordable materials used to build the fetal model (vaginal swabs, styrofoam ball, and putty) and the uterine model (collapsible water bottle). Required personnel roles included surgeon, surgical assistant, and facilitator. A standardized rubric was used to evaluate learners' mastery of procedural learning objectives, and a pre- and postsimulation assessment measured learners' knowledge and confidence before and after the activity. Consistency between iterations was maintained with use of standardized prompts and lectures. Total time for the activity, including setup and debrief, was 1 hour. **Results:** Eighteen residents, medical students, and attendings participated in the simulation, and 100% completed the assessment. There was a demonstrated improvement in clinical knowledge of D&E steps (56% presimulation vs. 94% postsimulation, $p < .001$) and increased surgical confidence in performing D&Es after participating in the simulation (28% presimulation vs. 89% postsimulation, $p < .001$). The participants with prior clinical experience in performing D&Es rated the fetal, uterine, and cervical models as realistic components in the simulation. **Discussion:** A gap now exists in access to clinical D&E training. This low-fidelity D&E simulation is a training tool that can fill this gap and improve learners' familiarity with this surgical procedure.

Keywords
Abortion Access, Abortion Training, Dilation and Evacuation, Gynecologic Surgery, Women's Health, Clinical/Procedural Skills Training, OB/GYN, Simulation

Educational Objectives

By the end of this activity, learners will be able to:

1. Perform the procedural steps of a dilation and evacuation (D&E) on a model.
2. Demonstrate clinical understanding of the variety of surgical equipment required to perform a D&E.
3. Discuss preoperative considerations, postoperative considerations, and postoperative complications that accompany D&Es.
4. Demonstrate improvement in knowledge and familiarity in performing the procedural steps of a D&E.

Citation:
Hunkler K, Boedeker D, Gill E, et al.. Second trimester abortion: a dilation and evacuation simulation for gynecologic surgery and obstetrics residents. *MedEdPORTAL*. 2025;21:11489.
https://doi.org/10.15766/mep_2374-8265.11489

Introduction

In 2021, 6.5% of abortions were performed after 13 weeks' gestation, the majority of which (93.6%) were surgically managed with a procedure known as a dilation and evacuation (D&E).[1,2] Patients seek abortions in the second trimester for various reasons, such as personal decision, intrauterine fetal demise, significant genetic anomalies, and life-threatening maternal complications.[3,4] The American College of Obstetricians and Gynecologists (ACOG) recommends that all gynecologic surgery and obstetrics (GS&O) residency programs provide comprehensive abortion training with an opt-out option.[5,6] Additionally, the Council on Resident Education in Obstetrics and Gynecology (CREOG) highlights the importance of this skill in its objective for residents to understand and perform D&Es.[7]

Despite the ACOG recommendation, the CREOG objective, and the Accreditation Council for Graduate Medical Education

Copyright © 2025 Hunkler et al. This is an open-access publication distributed under the terms of the Creative Commons Attribution license.

(ACGME) requirement that all GS&O residency programs offer abortion training, only 51% of program directors and 54% of trainees state that their programs offer routine training.[8,9] Furthermore, following the *Dobbs v Jackson Women's Health Organization* ruling,[10] it is estimated that up to 48% of GS&O residency programs are in states that are certain or likely to restrict abortion access.[11] As a result of these legal restrictions, trainees and providers are almost assured of having an increasingly difficult time developing and honing their abortion skills, specifically, performing D&Es.

To help address this gap in training, we created a low-cost, low-fidelity D&E simulation to train GS&O residents. Simulation training has been shown to improve provider confidence and ability, particularly for rare procedures.[12-14] GS&O skill simulations are commonly used, but there have been few simulations created for D&E skill development.[15-17] Many of the previously documented D&E simulations provide realistic models to imitate the tactile feel of the procedure, but accurate resemblance of the simulated fetus, calvarium, amniotic sac, and placental models varies.[18-20] ACOG has one of the more robust low-fidelity models available on its website; however, most of the components of the fetal model are food items that necessitate a onetime use.[21] A more recent publication in 2023 in *MedEdPORTAL* demonstrates a high-fidelity D&E simulation with postprocedural hemorrhage management; however, this simulation requires a high-resource center with a birthing mannequin, fake blood, and other simulation equipment, which may limit generalizability to lower-resourced residency programs.[22]

Our D&E simulator aims to create a realistic and low-cost option for GS&O residency programs to preserve second trimester abortion training among resident physicians. Unlike previously described resources, our simulation is unique in that it allows for multiple iterations utilizing the same low-cost equipment. This simulation builds upon our previously published quality improvement project that led to the creation of a standardized D&E checklist and virtual lecture.[23] The checklist and lecture supplement the simulation's teaching of the procedural steps of a D&E with a discussion of preoperative and postoperative considerations and complications, which aligns with the ACGME's graduation goal for GS&O residents: "Performs surgical uterine evacuation on patients with complex comorbidities and manages complications."[24] We conducted both the previous project (standardized checklist and lecture) and the simulation presented here in the military health care system. The Hyde Amendment has long restricted abortion care within the military, only allowing the procedures at military facilities with federal funds in cases of rape, incest, or life endangerment.[25] As a result, our GS&O residents have limited exposure to abortion training at our home institution, but they may complete family planning rotations at civilian hospitals. The simulation we describe here utilizes easy-to-access supplies and provides training materials that can be easily adopted by other programs that face similar training restrictions.

Methods

Development

Our GS&O residency program had 19 residents, all of whom were active-duty military service members and completed most of their clinical rotations at Walter Reed National Military Medical Center. Our previous quality improvement project informed us that implementing a virtual lecture and checklist at most military GS&O residency programs led to an improvement in trainees' self-reported comfort and knowledge in all procedural aspects of D&Es.[23] Therefore, the target learning population for our simulation was all available learners at our home institution during didactics as part of the obstetrics simulation curriculum in order to include all learners, most of whom had limited D&E procedural experience, regardless of whether they had had exposure to abortion training. No prerequisite knowledge, prereading, or prior family planning rotation was required for learners to participate in our simulation. No prior experience in performing D&Es was required of the facilitator due to the use of a standardized checklist and lecture.

Equipment/Environment

The simulation was performed in a large conference room and required one large table for three simulation models. The following equipment was needed to perform the simulation:

- Fetal model: To build the model fetus, we used one tin of Thinking Putty, a two- to three-centimeter Styrofoam ball, and three vaginal swabs per model. First, we broke down the three vaginal swabs into five separate four-centimeter pieces. We then molded a quarter-sized amount of Thinking Putty around each piece of the vaginal swab to model the fetal upper extremities, lower extremities, and thorax. Next, we pierced the Styrofoam ball with one of the vaginal swab pieces to simulate the fetal calvarium and spine. We attached the fetal calvarium and spine to the thorax and extremities using putty and additional vaginal swab pieces for support when needed. (This process is explained in Appendix A.) The fetal model could use longer or shorter pieces with more or less putty to stimulate varying gestational ages based on year-level objectives. Larger or

Copyright © 2025 Hunkler et al. This is an open-access publication distributed under the terms of the Creative Commons Attribution license.

smaller foam spheres could also simulate differently sized calvariums.

- Second trimester uterine model: To create the uterine model, we cut a seam at the top of a collapsible water container furthest from the spout. We then took a one-meter segment of Coban wrap and folded it in until we had created an eight-centimeter segment and placed it inside the water container with double-sided tape to mimic the placenta. We used a Toomey syringe to fill a water balloon with 50 milliliters of tap water and then placed a water balloon through the water container, wedging it into the opening of the water container to simulate the amniotic sac. Next, we inserted the fetal model into the uterine model and filled the uterine wall about a third of the way full with fluid to simulate amniotic fluid.
- Stand for model: We built a stand to support the model by taking a medium-sized cardboard box and completely breaking it down. Then, we folded the cardboard box in half along its natural bend point. We created a crease in the side of the box, several inches from the box's natural crease point, which formed an additional side. We rotated this additional side to lie on top of the other side, creating a triangular stand (Appendix A).
- Surgical instruments: These instruments included ovum forceps (either Sopher or Bierer), tenaculum, and under-buttocks drape to collect fluid (Appendix A).
- Facilitator prompts and simulation procedure steps (Appendix B).
- Standardized learner grading rubric (Appendix C).
- D&E standardized debrief lecture and checklist (Appendix D).
- D&E standardized debrief lecture and checklist speaker notes (Appendix E).
- Pre- and postsimulation assessment (Appendix F).
- Simulation video (Appendix G).
- Facilitator sequence of events (Appendix H).

Personnel

To successfully complete this simulation, the following personnel were required:

- GS&O learner primary surgeon
- GS&O learner surgical assistant
- Facilitator

Two learners were placed at each simulation station, with one learner serving as the primary surgeon and one learner serving as the surgical assistant. The learners switched roles during subsequent iterations.

Implementation

Prior to the simulation, two resident physicians and one fellow physician set up the simulation with three uterine models and three fetal models. Surgical instruments were also gathered at this time. This setup took approximately 10 minutes.

Prior to participation, all learners were asked to complete an optional anonymous hard-copy presimulation assessment (Appendix F). The presimulation assessment was printed out ahead of time and distributed at the start of the 30-minute session. Next, all learners viewed a 7-minute instructional video (Appendix G) that taught background statistics about D&Es, explained how to build the simulation, and demonstrated a learner performing the steps of a D&E using the simulator. The learners were divided among the three stations, with two learners and one facilitator per station. The facilitator engaged in a 5-minute discussion regarding preoperative considerations with all primary and assistant surgeons following standard prompts (Appendix B) before starting the simulation.

The learner designated as the primary surgeon started the simulation by verbalizing the preprocedural bimanual exam, operating room setup, and desired cervical dilation (about 2 minutes per learner). Using the surgical instruments, the learner then performed the steps of a D&E (5-10 minutes per learner; Appendix B). The learner ended the simulation by verbalizing necessary postprocedural steps, including accounting for fetal parts and assessing the cervix. The simulation ended when the learner vocalized that they had completed their postprocedural assessment. Each learner was assessed by the facilitator on their ability to perform the steps of a D&E using a standardized rubric (Appendix C). The primary and assistant surgeons then switched roles, and the simulation was repeated with a rebuilt fetal model that molded the putty over the styrofoam ball and vaginal swabs Following the simulation, the facilitator engaged in a 5-minute discussion of postoperative considerations and complications with all primary and assistant surgeons using standard prompts (Appendix B).

The simulation lasted 20-25 minutes total for two iterations, allowing all residents to serve as surgeons for one iteration and surgical assistants for another iteration. The learners then debriefed for 10 minutes as a group with the facilitator (Appendices D and E) and were given an optional postsimulation assessment (Appendix F). We were able to assess six learners in about 30-35 minutes, with two learners per uterine model. The sequence of events is outlined in Appendix H.

Copyright © 2025 Hunkler et al. This is an open-access publication distributed under the terms of the Creative Commons Attribution license.

Debriefing
The debrief began with the learners describing the simulation and sharing their reflections on their performance. The facilitator reiterated the initial learning objectives and led the learners through a standardized 10-minute PowerPoint presentation that discussed our previously published standardized D&E checklist as well as preoperative and postoperative considerations important to D&Es (Appendices D and E). Learners were given opportunities to ask questions and seek clarification. Following the debrief, learners were given the opportunity to complete an optional anonymous hard-copy postsimulation assessment (Appendix F).

Assessment
The facilitator assessed each learner's familiarity with performing the simulated D&E procedure using a standardized rubric (Appendix C). Scoring was based on completion of each item, even if completion required prompting of the learner by the facilitator.

All learner participants were invited to complete optional hard-copy presimulation and postsimulation assessments. A team of resident, fellow, and attending physicians with clinical experience in D&Es created the assessments with final approval from team members with hospital privileges to perform D&Es. The presimulation assessment (Appendix F) included questions about learner demographics, D&E experience, and future intent to offer D&Es, as well as questions regarding learners' knowledge and comfort in performing D&Es scored on a 5-point Likert scale (1 = *Strongly Disagree,* 2 = *Disagree,* 3 = *Neutral,* 4 = *Agree,* 5 = *Strongly Agree*). The postsimulation assessment (Appendix F) asked participants about their impression of the flow of the simulation, as well as the realistic nature of the fetal model, cervical model, and uterine model. The postsimulation assessment also included the same knowledge and comfort questions about performing D&Es as the presimulation assessment using the same 5-point Likert scale. All participants were given a stapled packet of both the pre- and postassessment surveys at the beginning of the activity. This allowed for anonymity and pairing of responses. Paired presimulation and postsimulation assessment responses were compared using a Wilcoxon signed rank test. Assessment of the simulation was reviewed and deemed exempt by our institutional review board (protocol number DBS.2023.595).

Results

Eighteen individuals completed our simulation, with learner participants including residents, medical students, and one attending physician (Table 1). All participants completed the pre- and postlearning assessments, equating to a 100% response rate. Prior to the simulation, a minority of learners felt comfortable with their current level of knowledge, experience, and training in performing D&Es (agree or strongly agree, 28%). Similarly, most respondents had never completed a family planning rotation (83%) and had never performed a D&E (78%). However, most participants planned to offer D&Es in their future practice (94%).

Table 1. Participants' Demographic Information ($N = 18$)

Item[a]	No.	%
What is your current level of training?		
Medical student	5	28
PGY 1	5	28
PGY 2	3	17
PGY 3	2	11
PGY 4	2	11
Attending	1	6
I feel comfortable with my current knowledge, experience, and training regarding D&E.		
Strongly disagree	5	28
Disagree	5	28
Neutral	3	17
Agree	4	22
Strongly agree	1	6
Have you completed a family planning rotation or another rotation focused on teaching D&E?		
Yes	3	17
No	15	83
How many D&Es have you performed?		
None	14	78
<5	2	11
5-10	0	0
10-20	0	0
>20	2	11
Do you intend to offer D&E procedures in your future practice?		
Yes	17	94
No	1	6

Abbreviation: D&E, dilation and evacuation.
[a]Completed presimulation.

Participants were asked about knowledge of the surgical steps of D&Es and their confidence in performing D&Es before and after participating in the simulation, with results utilizing a 5-point Likert scale (1 = *Strongly Disagree,* 2 = *Disagree,* 3 = *Neutral,* 4 = *Agree,* 5 = *Strongly Agree*; Figure). Participants' overall knowledge increased by approximately 1.6 points on the Likert scale ($p < .001$). Similarly, confidence in performing D&Es increased by 1.5 points on the Likert scale following the simulation ($p < .001$; Table 2).

Five of the 18 participants had performed D&Es previously. Of those five participants, all reported that the simulation was realistic compared to prior D&Es they had performed (agree or strongly agree, 100%). They also reported that the fetal model, cervical model, and tactile sensation in the simulation were realistic (agree or strongly agree, 100%). The majority of those who had previously completed a D&E stated the compression of

Copyright © 2025 Hunkler et al. This is an open-access publication distributed under the terms of the Creative Commons Attribution license.

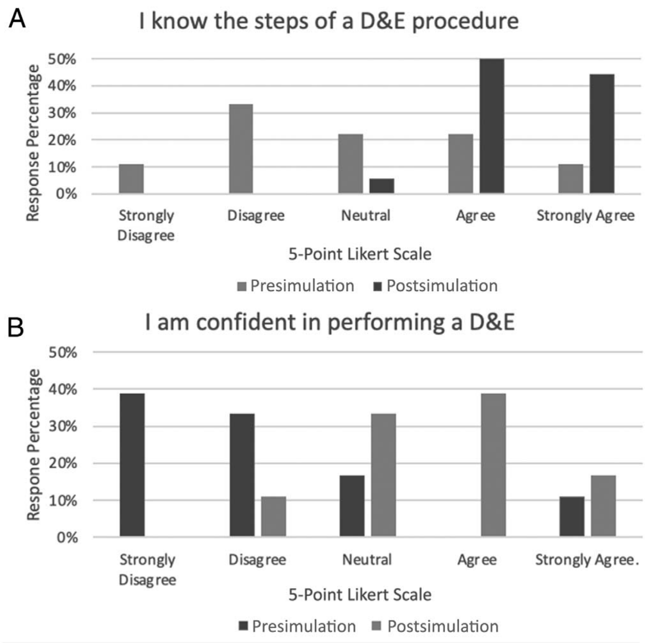

Figure. Presimulation and postsimulation assessment responses ($N = 18$). Descriptive modeling depicts overall trends in responses for knowledge and confidence. A: Knowledge of steps in a D&E procedure. B: Confidence in performing a D&E. Abbreviation: D&E, dilation and evacuation.

the calvarium and uterine model were realistic (agree or strongly agree, 80%).

All participants reported that this simulation enhanced their understanding of D&Es (agree or strongly agree, 100%; Table 3). Most participants felt the simulation's flow and sequence were appropriate (agree, strongly agree, 89%). However, about half of participants felt they still needed additional training and preparation before performing D&E (agree or strongly agree, 56%).

Discussion

We implemented a low-cost, reproducible simulation for second trimester D&E that could be used for multiple iterations. This simulation is unique because it incorporates an instructional video, accessible materials, standardized prompts, and a scripted lecture to maximize all educational adjuncts to suit multiple

Table 2. Participants' Self-Reported Knowledge and Confidence in Performing Dilation and Evacuations Before and After Simulation ($N = 18$)

Item[a]	Presimulation *M*	Postsimulation *M*	*M* Difference[b]	*p*
Knowledge	2.9	4.6	1.6	<.001
Confidence	2.1	3.6	1.5	<.001

[a]Rated on a 5-point Likert scale (1 = *Strongly Disagree*, 5 = *Strongly Agree*).
[b]Means compared using Wilcoxon signed rank test.

Table 3. Participants' Overall Evaluation of the Simulation ($N = 18$)

Item[a]	No.	%
The simulation's sequence and flow were appropriate.		
Strongly disagree	2	11
Disagree	0	0
Neutral	0	0
Agree	6	33
Strongly agree	10	56
I feel I need additional training and preparation before performing a D&E.		
Strongly disagree	1	6
Disagree	2	11
Neutral	5	28
Agree	7	39
Strongly agree	3	17
I feel that this simulation enhanced my understanding of a D&E.		
Strongly disagree	0	0
Disagree	0	0
Neutral	0	0
Agree	5	28
Strongly agree	13	72

Abbreviation: D&E, dilation and evacuation.
[a]Completed postsimulation.

learning styles. Our results showed significant differences in knowledge and confidence in performing procedural aspects of D&Es before and after participating in the simulation. Among residents who had clinical experience with D&Es, the uterine, fetal, and cervical models were all deemed realistic. Additionally, most participants planned to offer D&E in their future clinical practice, and all participants felt the simulation helped enhance their understanding of D&E.

Building upon prior scholarly work was essential in ensuring an evidence-based simulation would be carried out. The instructional video, facilitator prompts, and flow of the D&E simulation used knowledge gained during our previous quality improvement project that resulted in the evidence-based checklist and lecture. Development of the D&E checklist and lecture involved gathering high-quality evidence and using a team of experts with subspecialty training in family planning to vet the checklist for appropriate clinical practice.[23] Both scholarly projects were presented to a military audience, which may have limited generalizability, though a lack of exposure to D&Es is expected to become more commonplace in the civilian environment, and therefore, the activity likely does not require modification for use in other settings.

This activity was presented during our residency program's required academics. While learners' participation in the simulation was required, they were informed that the pre- and postlearning assessment was optional; however, printed copies were readily available. It is possible the required nature of this activity influenced participants' views on the success of the simulation

Copyright © 2025 Hunkler et al. This is an open-access publication distributed under the terms of the Creative Commons Attribution license.

and thereby led to a bias reflected in our results. Conversely, performing this activity during academics allowed us to optimize the number of recruited learners participating in the optional pre- and postlearning assessment. Additionally, given the ACGME and CREOG educational objectives related to D&Es, the inclusion of this simulation as a required activity in academics was appropriate. To facilitate different participant learning styles, a strength of this project is its use of multiple teaching adjuncts, such as an instructional video, standard facilitator prompts, hands-on tactile model, and scripted debrief lecture. The various instructional materials allow inexperienced facilitators to run the simulation, which is a strength but also an inherent limitation when considering real clinical application of the model. Another limitation is the small number of participants, which is reflective of most GS&O residency programs and therefore an inherent limitation of educational activities in this field. Our project specifically included mostly novice learners, with only a few participants with prior D&E clinical experience, which may have inhibited assessment of the clinical accuracy of the model. While the actual heterogeneous audience with many novice learners differed from the intended audience of mainly resident learners, our actual audience may have benefited even more from the simulation as their first exposure to D&E training, but translation from simulation to operation was not tested.

We plan to expand participation among learners with prior D&E clinical experience in future iterations to further evaluate and validate the realistic nature of this low-fidelity model. This simulation could also be expanded to other abortion providers such as nurse practitioners and family medicine physicians given its comprehensive nature in reviewing the subject matter. Our participants included a large portion of medical students, many of whom had limited D&E exposure and procedural skills. The fact that the simulation demonstrated an improvement in comfort with D&Es among this cohort suggests it can be used with other providers who may be unfamiliar with D&Es in order to teach them this important skill. Future facilitators may wish to expand the number of questions included in the pre- and postsimulation assessments to include comfort handling complications with D&Es, as the provided assessments omit questions specifically pertaining to this topic. In a similar vein, depending on the learners participating in the simulation, facilitators may wish to invert the sequence of events and start with the discussion prior to the simulation in order to teach preoperative considerations and operative steps.

Simulation and other supplements for clinical experience are of the utmost importance in today's political climate. Preserving patients' access to care starts with proper training. We hope that our simulation can serve as a valuable tool for educators everywhere who desire to introduce this surgical skill to new learners. For more experienced learners, a logical next step is a high-fidelity model with more clinical applications than our low-fidelity model.

Appendices

A. Materials and Instructions.docx
B. Facilitator Guide.docx
C. Learner Grading Rubric.docx
D. Simulation Debrief.pptx
E. Speaker Notes for Debrief.docx
F. Pre- and Postsimulation Assessment.docx
G. Simulation Video.mov
H. Facilitator Sequence of Events.docx

All appendices are peer reviewed as integral parts of the Original Publication.

Kiley Hunkler, MD, MSt, MSc: Fellow, Department of Gynecologic Surgery and Obstetrics, Walter Reed National Military Medical Center; Fellow, Reproductive Endocrinology and Infertility, National Institute of Child Health and Human Development, National Institutes of Health; ORCID: https://orcid.org/0009-0002-6292-712X

David Boedeker, DO, MHA: Fourth-Year Resident, Department of Gynecologic Surgery and Obstetrics, Walter Reed National Military Medical Center

Elizabeth Gill, MD: Second-Year Resident, Department of Gynecologic Surgery and Obstetrics, Walter Reed National Military Medical Center

Katelyn Simpkins, MD: Third-Year Resident, Department of Gynecologic Surgery and Obstetrics, Walter Reed National Military Medical Center

Katerina Shvartsman, MD: Associate Professor, Department of Gynecologic Surgery and Obstetrics, Uniformed Services University of the Health Sciences F. Edward Hébert School of Medicine

Jill Brown, MD, MPH, MHS: Associate Professor, Department of Gynecologic Surgery and Obstetrics, Uniformed Services University of the Health Sciences F. Edward Hébert School of Medicine

Sara Drayer, MD: Fellow, Department of Gynecologic Surgery and Obstetrics, Walter Reed National Military Medical Center

Disclosures
None to report.

Funding/Support
None to report.

Copyright © 2025 Hunkler et al. This is an open-access publication distributed under the terms of the Creative Commons Attribution license.

Ethical Approval
The Uniformed Services University Human Research Protections Program deemed further review of this project not necessary.

Disclaimer
The identification of specific products or scientific instrumentation is considered an integral part of the scientific endeavor and does not constitute endorsement or implied endorsement on the part of the author(s), Department of Defense, or any component agency. The opinions and assertions expressed herein are those of the authors and do not reflect the official policy or position of the Uniformed Services University of the Health Sciences, the Department of the Army, the Department of the Air Force, the Department of the Navy, or the Department of Defense.

References

1. Kortsmit K, Nguyen AT, Mandel MG, et al. Abortion surveillance—United States, 2021. *MMWR Surveill Summ*. 2023;72(9):1-29. https://doi.org/10.15585/mmwr.ss7209a1
2. Drey EA, Foster DG, Jackson RA, Lee SJ, Cardenas LH, Darney PD. Risk factors associated with presenting for abortion in the second trimester. *Obstet Gynecol*. 2006;107(1):128-135. https://doi.org/10.1097/01.AOG.0000189095.32382.d0
3. Shaffer BL, Caughey AB, Norton ME. Variation in the decision to terminate pregnancy in the setting of fetal aneuploidy. *Prenat Diagn*. 2006;26(8):667-771. https://doi.org/10.1002/pd.1462
4. Practice bulletin no. 135: second-trimester abortion. *Obstet Gynecol*. 2013;121(6):1394-1406. https://doi.org/10.1097/01.AOG.0000431056.79334.cc
5. Increasing access to abortion: ACOG committee opinion, number 815. *Obstet Gynecol*. 2020;136(6):e107-e115. https://doi.org/10.1097/AOG.0000000000004176
6. Committee opinion no. 612: abortion training and education. *Obstet Gynecol*. 2014;124(5):1055-1059. https://doi.org/10.1097/01.AOG.0000456327.96480.18
7. *Council on Resident Education in Obstetrics and Gynecology (CREOG) Educational Objectives: Core Curriculum in Obstetrics and Gynecology*. 13th ed. American College of Obstetricians and Gynecologists; 2022. Accessed December 30, 2024. https://www.acog.org/-/media/project/acog/acogorg/files/creog/creog-educational-objectives-13th-edition.pdf?rev=53bf30ec0f144132a8d81d81549b2476
8. Eastwood KL, Kacmar JE, Steinauer J, Weitzen S, Boardman LA. Abortion training in United States obstetrics and gynecology residency programs. *Obstet Gynecol*. 2006;108(2):303-308. https://doi.org/10.1097/01.AOG.0000224705.79818.c9
9. Turk JK, Preskill F, Landy U, Rocca CH, Steinauer JE. Availability and characteristics of abortion training in US ob-gyn residency programs: a national survey. *Contraception*. 2014;89(4):271-277. https://doi.org/10.1016/j.contraception.2013.12.002
10. *Dobbs v Jackson Women's Health Organization,* 597 US 215 (2022).
11. Vinekar K, Karlapudi A, Nathan L, Turk JK, Rible R, Steinauer J. Projected implications of overturning *Roe v Wade* on abortion training in U.S. obstetrics and gynecology residency programs. *Obstet Gynecol*. 2022;140(2):146-149. https://doi.org/10.1097/AOG.0000000000004832
12. Aggarwal R, Ward J, Balasundaram I, Sains P, Athanasiou T, Darzi A. Proving the effectiveness of virtual reality simulation for training in laparoscopic surgery. *Ann Surg*. 2007;246(5):771-779. https://doi.org/10.1097/SLA.0b013e3180f61b09
13. Barsuk JH, McGaghie WC, Cohen ER, Balachandran JS, Wayne DB. Use of simulation-based mastery learning to improve the quality of central venous catheter placement in a medical intensive care unit. *J Hosp Med*. 2009;4(7):397-403. https://doi.org/10.1002/jhm.468
14. Barsuk JH, McGaghie WC, Cohen ER, O'Leary KJ, Wayne DB. Simulation-based mastery learning reduces complications during central venous catheter insertion in a medical intensive care unit. *Crit Care Med*. 2009;37(10):2697-2701. https://doi.org/10.1097/00003246-200910000-00003
15. Nitsche JF, Fino NF, Palomo JM, Perdomo AP, Brost BC. Effectiveness of a labor cervical exam model in family medicine and OB-GYN residents. *Fam Med*. 2017;49(5):384-387.
16. Mannella P, Palla G, Cuttano A, Boldrini A, Simoncini T. Effect of high-fidelity shoulder dystocia simulation on emergency obstetric skills and crew resource management skills among residents. *Int J Gynaecol Obstet*. 2016;135(3):338-342. https://doi.org/10.1016/j.ijgo.2016.06.023
17. Chong W, Downing K, Leegant A, Banks E, Fridman D, Downie S. Resident knowledge, surgical skill, and confidence in transobturator vaginal tape placement: the value of a cadaver laboratory. *Female Pelvic Med Reconstr Surg*. 2017;23(6):392-400. https://doi.org/10.1097/SPV.0000000000000458
18. Baldwin MK, Chor J, Chen BA, Edelman AB, Russo J. Comparison of 3 dilation and evacuation technical skills models. *J Grad Med Educ*. 2013;5(4):662-664. https://doi.org/10.4300/JGME-D-13-00049.1
19. York SL, McGaghie WC, Kiley J, Hammond C. Implementation and evaluation of a dilation and evacuation simulation training curriculum. *Contraception*. 2016;93(6):545-550. https://doi.org/10.1016/j.contraception.2016.02.002
20. York SL, Migas S, Haag J, et al. Creation and initial assessment of a second-trimester uterine model. *Simul Healthc*. 2014;9(3):199-202. https://doi.org/10.1097/SIH.0000000000000042
21. Curriculum: Simulations Working Group—second-trimester dilation and evacuation. American College of Obstetrics and Gynecologists. Accessed November 2022. https://www.acog.org/education-and-events/simulations/scog019
22. Schwartz LN, Pelletier A, Goldberg AB, et al. Second-trimester dilation and evacuation: a simulation-based team training

Copyright © 2025 Hunkler et al. This is an open-access publication distributed under the terms of the Creative Commons Attribution license.

curriculum. *MedEdPORTAL*. 2023;19:11336. https://doi.org/10.15766/mep_2374-8265.11336

23. Hunkler K, Pekny C, Boedeker D, Owens A, Wiersma D, Drayer S. Strengthening abortion training: a dilation and evacuation checklist in military resident education. *Mil Med*. 2024;189(11-12):2402-2407. https://doi.org/10.1093/milmed/usad491

24. Accreditation Council for Graduate Medical Education. *Obstetrics and Gynecology Milestones*. Accreditation Council for Graduate Medical Education; 2021. Accessed December 30, 2024. https://www.acgme.org/globalassets/pdfs/milestones/obstetricsandgynecologymilestones.pdf

25. Kamarck KN, Mendez BHP. *FY2023 NDAA: Military Abortion Policies*. Congressional Research Service; 2023. IN11960 version 5. Accessed December 30, 2024. https://crsreports.congress.gov/product/pdf/IN/IN11960

Received: March 27, 2024
Accepted: November 14, 2024
Published: January 21, 2025

Copyright © 2025 Hunkler et al. This is an open-access publication distributed under the terms of the Creative Commons Attribution license.
